# Cyclic multiplex fluorescent immunohistochemistry and machine learning reveal distinct states of astrocytes and microglia in normal aging and Alzheimer’s disease

**DOI:** 10.1186/s12974-022-02383-4

**Published:** 2022-02-02

**Authors:** Clara Muñoz-Castro, Ayush Noori, Colin G. Magdamo, Zhaozhi Li, Jordan D. Marks, Matthew P. Frosch, Sudeshna Das, Bradley T. Hyman, Alberto Serrano-Pozo

**Affiliations:** 1 Departamento de Bioquímica y Biología Molecular, Facultad de Farmacia, Universidad de Sevilla, 41012 Sevilla, Spain; 2 Instituto de Biomedicina de Sevilla (IBiS)-Hospital Universitario Virgen del Rocío/CSIC/Universidad de Sevilla, 41013 Sevilla, Spain; 3 Harvard College, Boston, MA 02138 USA; 4 Department of Neurology, Massachusetts General Hospital, Boston, MA 02114 USA; 5 Department of Pathology, Massachusetts General Hospital, Boston, MA 02114 USA; 6 MassGeneral Institute for Neurodegenerative Disease, 114 16th Street, Charlestown, MA 02129 USA; 7 Massachusetts Alzheimer’s Disease Research Center, Charlestown, MA 02129 USA; 8 Harvard Medical School, Boston, MA 02115 USA

**Keywords:** Alzheimer’s disease, Amyloid plaques, Astrocytes, Immunohistochemistry, Microglia, Neurofibrillary tangles, Neuropathology, Tau

## Abstract

**Background:**

Astrocytes and microglia react to Aβ plaques, neurofibrillary tangles, and neurodegeneration in the Alzheimer’s disease (AD) brain. Single-nuclei and single-cell RNA-seq have revealed multiple states or subpopulations of these glial cells but lack spatial information. We have developed a methodology of cyclic multiplex fluorescent immunohistochemistry on human postmortem brains and image analysis that enables a comprehensive morphological quantitative characterization of astrocytes and microglia in the context of their spatial relationships with plaques and tangles.

**Methods:**

Single FFPE sections from the temporal association cortex of control and AD subjects were subjected to 8 cycles of multiplex fluorescent immunohistochemistry, including 7 astroglial, 6 microglial, 1 neuronal, Aβ, and phospho-tau markers. Our analysis pipeline consisted of: (1) image alignment across cycles; (2) background subtraction; (3) manual annotation of 5172 ALDH1L1+ astrocytic and 6226 IBA1+ microglial profiles; (4) local thresholding and segmentation of profiles; (5) machine learning on marker intensity data; and (6) deep learning on image features.

**Results:**

Spectral clustering identified three phenotypes of astrocytes and microglia, which we termed “homeostatic,” “intermediate,” and “reactive.” Reactive and, to a lesser extent, intermediate astrocytes and microglia were closely associated with AD pathology (≤ 50 µm). Compared to homeostatic, reactive astrocytes contained substantially higher GFAP and YKL-40, modestly elevated vimentin and TSPO as well as EAAT1, and reduced GS. Intermediate astrocytes had markedly increased EAAT2, moderately increased GS, and intermediate GFAP and YKL-40 levels. Relative to homeostatic, reactive microglia showed increased expression of all markers (CD68, ferritin, MHC2, TMEM119, TSPO), whereas intermediate microglia exhibited increased ferritin and TMEM119 as well as intermediate CD68 levels. Machine learning models applied on either high-plex signal intensity data (gradient boosting machines) or directly on image features (convolutional neural networks) accurately discriminated control vs. AD diagnoses at the single-cell level.

**Conclusions:**

Cyclic multiplex fluorescent immunohistochemistry combined with machine learning models holds promise to advance our understanding of the complexity and heterogeneity of glial responses as well as inform transcriptomics studies. Three distinct phenotypes emerged with our combination of markers, thus expanding the classic binary “homeostatic vs. reactive” classification to a third state, which could represent “transitional” or “resilient” glia.

**Supplementary Information:**

The online version contains supplementary material available at 10.1186/s12974-022-02383-4.

## Background

Reactive astrocytes and microglia are prominent features of the Alzheimer’s disease (AD) brain landscape, typically decorate dense-core neuritic amyloid-β (Aβ) plaques throughout the cortex, and represent a phenotypic change of existing homeostatic glial cells rather than proliferation of glial progenitors [1–6]. Reactive astrocytes have traditionally been depicted with immunohistochemistry for glial fibrillary acidic protein (GFAP), whereas reactive microglia have often been demonstrated with cluster differentiation 68 (CD68) or major histocompatibility complex II (MHC2, also known as HLA-DP-DQ-DR), but this classic approach obviates the complexity and heterogeneity of the changes that these glial cell types undergo in the vicinity of Aβ plaques and neurofibrillary tangles (NFTs) [7]. Although this complexity is currently emerging with the advent of single-nuclei and single-cell RNA sequencing (snRNA-seq and scRNA-seq) [8–14], one limitation of such methods is their lack of spatial information. While the spatial resolution of tissue-based spatial transcriptomics and proteomics techniques is rapidly improving [15, 16], methods to comprehensively phenotype brain cells at a single-cell resolution while preserving cellular spatial relationships with neuropathological lesions remain an urgent need.

In this study, we developed a novel protocol of cyclic multiplex fluorescent immunohistochemistry in formalin-fixed paraffin-embedded (FFPE) sections followed by a quantitative image analysis pipeline including machine learning, both of which allowed a thorough phenotyping of astrocytes and microglia in postmortem control (CTRL) and AD brains at single-cell resolution. Using this methodology, we tested the hypothesis that diverse astrocytic and microglial phenotypes populate the cerebral cortex of cognitively healthy and AD subjects, and that AD-associated glial reactions are heterogeneous. We identified at least three distinct phenotypes of both astrocytes and microglia, which we termed homeostatic, intermediate, and reactive, and that imply both functional gains and losses. We also demonstrate that reactive and, to a lesser extent, intermediate glial cells are spatially associated with AD pathological hallmarks. Finally, machine learning models classified astrocytes and microglia as CTRL vs. AD with high accuracy, suggesting that biomarker profiling of glia has diagnostic predictive value.

## Methods

### Brain specimens

Eight-micron-thick FFPE sections from the temporal lobe pole of CTRL (*n* = 7, age [mean ± SD] 86.0 ± 2.5 years, sex 4M/3F) and AD (*n* = 7, 76.7 ± 11.2 years, 3M/4F) donors were obtained from the Massachusetts Alzheimer’s Disease Research Center (MADRC) Brain Bank. AD subjects met the clinical and neuropathological criteria for AD with NIA-AA scores of A3B3C2 or A3B3C3 [17–19], whereas CTRL subjects were not demented and did not meet the neuropathological criteria for any neurodegenerative disease. Demographic, clinical, and pathological characteristics of these cases are described in Table S1: Additional file 1. All subjects or their next of kin had given written informed consent for the brain donation and the study was approved under the MADRC Brain Bank Institutional Review Board. The temporal pole was selected because the temporal association cortex in this region is an area of early and abundant Aβ plaque and NFT deposition in AD [2–5] (Figs. S1 and S2: Additional file 2).

### Cyclic multiplex fluorescent immunohistochemistry protocol

We performed multiplex fluorescent immunohistochemistry in single FFPE sections by combining the Opal method [20] and the tissue-based cyclic immunofluorescence (t-CyCIF) protocol developed by the Sorger laboratory [21–23]. Briefly, fluorescent immunohistochemistry was performed with primary antibodies and species-appropriate fluorescently-labeled secondary antibodies in each cycle as usual, but each imaging session was followed by antibody denaturation (by heating sections in the microwave as in the Opal method) and fluorescence quenching (as in the t-CyCIF protocol).

With this protocol, we assayed a total of 16 markers distributed across 8 cycles of immunohistochemistry, including 7 astrocytic markers (aldehyde dehydrogenase 1 L1 [ALDH1L1], glutamine synthetase [GS], the glutamate transporters excitatory amino acid transporter 1 [EAAT1, also known as GLAST-1] and excitatory amino acid transporter 2 [EAAT2, also known as GLT1], GFAP, vimentin [VIM], and chitinase 3-like protein 1 [YKL-40, also known as CHI3L1]), 6 microglial markers (CD68, ferritin [FTL], ionized calcium-binding adapter molecule 1 [IBA1], MHC2, transmembrane protein 119 [TMEM119], and the 18 kDa translocator protein [TSPO]), 1 neuronal marker (Hu-antigen C/D [HuC/D]), and Aβ and PHF1 (paired helical filament tau phosphorylated at Ser396 and Ser404) as markers of AD neuropathological changes (see Table S2: Additional file 1 for details).

**Fig. 1:**
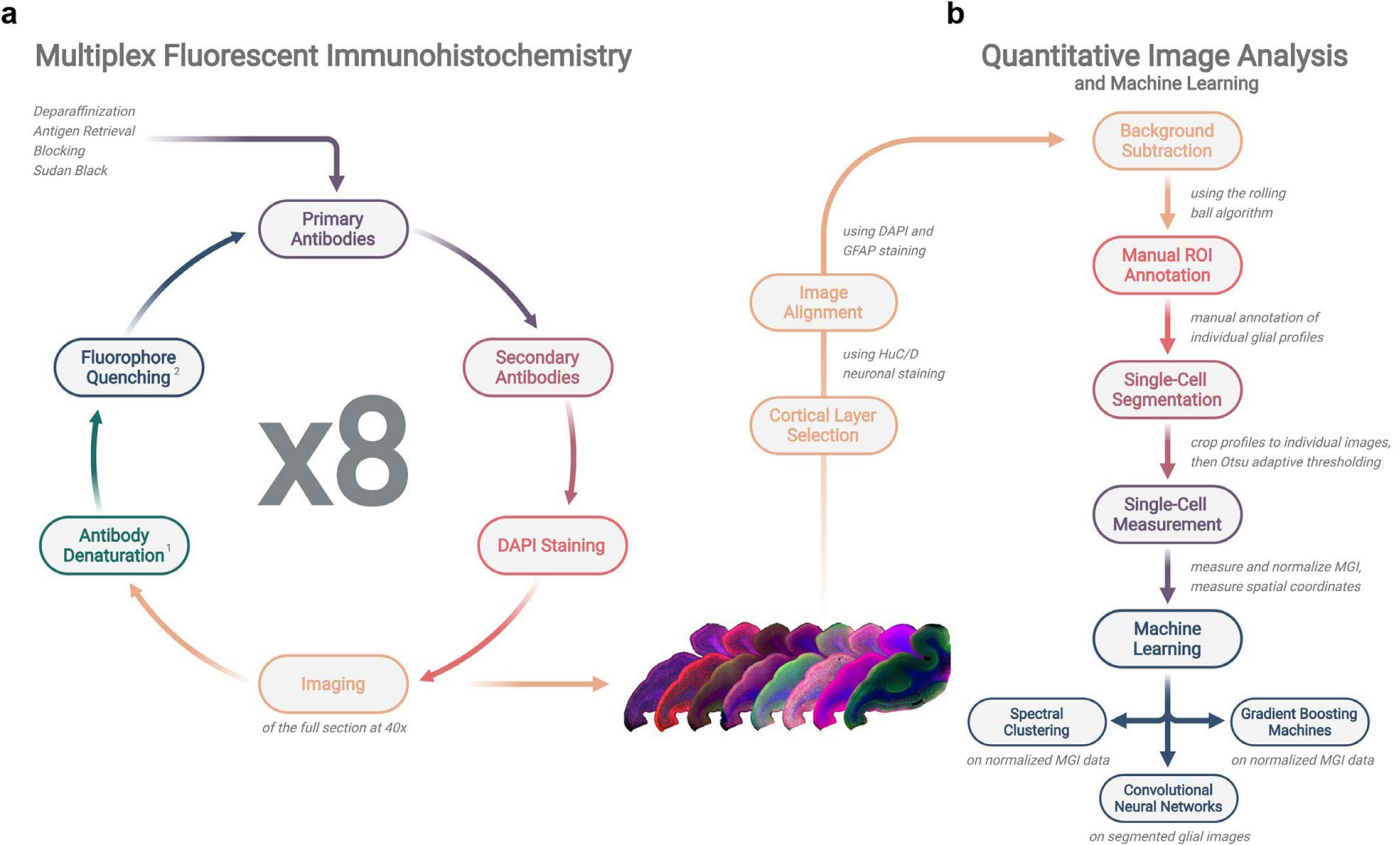
Workflow of cyclic multiplex fluorescent immunohistochemistry and machine learning-based quantitative image analysis. **a** Schematic of the cyclic multiplex fluorescent immunohistochemistry protocol, where (1) antibodies were denatured by microwave treatment in boiling citrate buffer for 20 min and (2) fluorophores were quenched by immersion in an oxidizing alkaline solution for 30 min (see details in Methods). **b** Flowchart of the quantitative image analysis and machine learning pipeline

Specifically, the protocol consisted of the following steps (Fig. 1a):

1. Dewaxing: Paraffin was cleared from tissue sections with xylenes (2 × 10 min) and sections were rehydrated in decreasing concentrations of ethanol (5 min in 100%, 5 min in 100%, 5 min in 95%, and 5 min in 70%), then transferred to distilled water (5 min).
2. Antigen retrieval: Sections were microwaved in boiling citrate buffer 0.01 M pH 6.0 with Tween 20 0.05% at 95 °C for 20 min followed by cooling at 4 °C for ∼ 45 min.
3. Washes in tris-buffered saline (TBS): 3 × 5 min.
4. Sudan black staining: Sections were immersed in 70% ethanol for 5 min, then treated with 2–3 drops of filtered Autofluorescence Eliminator Reagent (Millipore, #2160) for 5 min, then cleared with three 1 min serial immersions in ethanol 70%, followed by a 5 min wash in TBS.
5. Blocking: Sections were blocked with 10% normal donkey serum (NDS) in TBS for 1 h at room temperature (RT).
6. Primary antibody incubation: Primary antibodies were dissolved in 5% NDS in TBS at the appropriate concentrations and applied onto the sections overnight at 4 °C. Note that GFAP was included in all cycles to optimize the subsequent alignment of images obtained from individual cycles.
7. Washes in TBS: 2 × 10 min.
8. Secondary antibody incubation: Species-appropriate fluorescently-labeled secondary antibodies (Table S2: Additional file 1) were dissolved in 5% NDS in TBS at a 1:200 concentration and applied onto the sections for 2 h at RT.
9. Washes in TBS: 2 × 10 min.
10. Coverslipping of sections with a water-soluble mounting media containing DAPI (Fluoromount-G-DAPI, Southern Biotech, #0100-20). DAPI staining after each immunohistochemistry cycle facilitated downstream image alignment.
11. Imaging: Sections were scanned in a VS120 Olympus microscope (Olympus, Tokyo, Japan).
12. Removal of coverslips from sections by immersion in TBS with Tween20 0.05%.
13. Antibody denaturation and stripping: Sections were microwaved again in boiling citrate buffer 0.01 M pH 6.0 with Tween20 0.05% at 95 °C for 20 min followed by cooling at 4 °C for ∼ 45 min.
14. Washes with TBS: 3 × 5 min.
15. Fluorescence quenching: Sections were immersed in an oxidizing alkaline solution (NaHCO_3_ 0.1 M pH 11.2, H_2_O_2_ 3%) for 30 min at RT.
16. Washes with TBS: 3 × 5 min.
17. Repeat steps #5 to #16 for 7 more cycles. Note that we refrained from using formic acid as standard antigen retrieval pretreatment for Aβ due to the increased risk of tissue damage by the last cycle; however, the multiple rounds of antigen retrieval by microwaving in citrate buffer improved Aβ signal.

### Fluorescent microscopy

Sections were imaged on an Olympus VS120 virtual slide scanner with the 40× objective. To maintain consistency across sections, each marker was imaged at the same exposure time in all sections. The appropriate exposure times were decided based on pilot studies.

### Image alignment and segmentation

Image segmentation was performed with the FIJI distribution of the open-source Java-based image analysis program ImageJ [24, 25]. Unless otherwise indicated, all other computational and statistical analyses were performed in R (version 4.1.0). Our image segmentation pipeline consisted of the following steps (Fig. 1b):

1. Cortical layer selection: The six cortical layers were identified using the HuC/D neuronal staining; then, full-width 1 mm-long rectangular sections were cropped from each cortical layer to new images using cellSens image analysis software (Olympus, Tokyo, Japan). Because layer I astrocytes, also called subpial or interlaminar astrocytes, are substantially different in morphology and function relative to those from deeper layers, only layers II to VI were included in the analysis.
2. Image alignment across cycles: Layer-specific crops from all 8 cycles of immunohistochemistry (for a total of 17 channels: 16 primary antibodies plus DAPI) were aligned using the “Alignment module” of cellSens with the DAPI and GFAP images included in each round as reference.
3. Image blinding: Image files were each assigned a random alphanumeric code so that subsequent steps in image segmentation were blind to diagnosis (CTRL vs. AD) and donor identity.
4. Background subtraction: To minimize background differences across sections, we performed regional background subtraction in all images using the rolling ball algorithm in ImageJ with a radius of 200 pixels [21–23, 26]. In this algorithm, a local background value is determined for every pixel by averaging over a large radius (i.e., 200 pixels) around the pixel, and this value is then subtracted from the original image to remove spatial variations in the background intensities [27].
5. Manual annotation of individual regions of interest (ROIs): Single ALDH1L1+ and IBA1+ glial cell profiles, Aβ plaques, and PHF1+ NFTs were manually annotated using the box tool of the Visual Geometry Group (VGG) Image Annotator (VIA) [28]. ALDH1L1 and IBA1 were selected as constitutive markers of astrocytes and microglia because our prior stereology-based study in this same brain region showed no significant differences in the number of ALDH1L1+ astrocytes and IBA1+ microglial cells between CTRL and AD subjects [4]. ALDH1L1+ profiles consisted of astrocyte somas, including cell bodies and the stump of their primary branches, whereas IBA1+ profiles consisted of microglial cells (i.e., the cell body with all processes present) or processes (when the cell body was not visible). VIA annotations were subsequently parsed in R.
6. ROI segmentation: Manually annotated glial profiles, Aβ plaques, and PHF1+ NFTs were converted from parsed VIA annotations to ImageJ ROIs, and these single ROIs were segmented using adaptive Otsu thresholding in ImageJ [24, 25].
7. ROI measurement and pre-processing: We used mean gray intensity (MGI) as a proxy for protein expression level. Briefly, we measured the MGI for each astrocytic marker (EAAT1, EAAT2, GFAP [from the first cycle only], GS, TSPO, VIM, and YKL-40) within each ALDH1L1+ astrocyte profile and for each microglial marker (CD68, FTL, MHC2, TMEM119, and TSPO) within each IBA1+ microglia profile using the Measure tool in ImageJ. Next, as intensity-based measurements of protein expression often follow log-normal distributions [29], we applied a natural log transformation [21–23, 26] and obtained the z-scores of the log-transformed MGI values.
8. Distance between glial profiles and AD pathological lesions: For each glial profile, Aβ plaque, and PHF1+ NFT, the Measure tool in ImageJ also provided the spatial XY coordinates within its layer-specific crop. With these coordinates, we calculated the distance between each glial profile and the nearest Aβ plaque or PHF1+ NFT. Since plaques can be quite large, we subtracted the radius of each plaque (calculated from its circular area) from each raw distance measurement to obtain the exact distance between each glial profile and the nearest plaque edge (rather than the center of the nearest plaque).

### Machine learning

#### Spectral clustering

To identify diverse phenotypes or states of astrocytes and microglia within our highly multiplexed data set at single-cell resolution, we applied spectral clustering on the normalized MGI *z*-scores using the *SNFtool* package in R [30]. Spectral clustering is a graph-based, unsupervised machine learning approach which performs dimensionality reduction and partitions the data into network clusters based on similarity. Specifically, after creating a pairwise similarity matrix (where local affinity was defined by the scaled exponential similarity kernel as described in [30], albeit without the K-nearest neighbors sparsification), clustering was performed on the eigenvectors of the Laplacian of the similarity matrix. By recovering network connectivity, spectral clustering often outperforms traditional clustering algorithms (e.g., *k*-means, hierarchical) [31]. The optimal number of clusters was assessed with two independent methods: (1) the eigengap heuristic, which is based on the connectivity of the network as judged by the ranked eigenvalues of the Laplacian matrix, and (2) rotation cost, which is based on the structure of the eigenvectors of the Laplacian matrix. Both approaches discarded *n* = 1 and *n* = 4 clusters, and subsequent visual inspection of the heatmaps favored *n* = 3 over *n* = 2 clusters as the best fit for both astrocyte and microglia MGI data (not shown). After clustering ALDH1L1+ astrocytes and IBA1+ microglia profiles separately, the normalized MGI *z*-scores of the profiles of the resulting clusters were visualized via heatmaps (where rows were scaled to compare the relative expression between markers) and box and whisker plots.

#### Gradient boosting machine (GBM) models

To determine whether the combinations of astrocytic or microglial markers discriminate between CTRL and AD diagnoses as well as the relative contribution of each marker to the CTRL vs. AD classification, we built stochastic gradient boosting machine (GBM) models using the *caret* and *gbm* packages in R [32, 33]. GBM is a supervised machine learning method that constructs an ensemble of additive, shallow decision trees [34, 35]. Briefly, the data set of MGI *z*-scores was partitioned by stratified random sampling into training/cross-validation (80%) and hold-out test (20%) sets. Then, the hyperparameters of each model (i.e., number of trees and interaction depth) were optimized using 10-fold cross-validation on the training set. Next, the model performance was evaluated on the hold-out test set at the accuracy-maximizing thresholds and 95% confidence intervals were estimated by bootstrapping across 500 iterations.

Next, the astrocytic or microglial markers were ranked by their variable importance scores for the classification, which were obtained by computing the differences in prediction accuracy before and after permuting each predictor variable (i.e., each astrocytic or microglial marker), then summing the importance scores over each boosting iteration. Finally, analogous GBM models were constructed to discriminate between the glial states identified by spectral clustering (i.e., separate astrocytic or microglial profiles by phenotype rather than diagnosis) with the goal of determining the most relevant markers for this phenotypic classification.

#### Convolutional neural networks (CNNs)

*Model architecture* To investigate whether our highly multiplexed images of astrocytes and microglia can discriminate between CTRL and AD based not only on marker signal intensity (i.e., log-normalized MGI z-scores) but by all image features (e.g., pixel subcellular localization, cellular morphology, etc.), we designed deep learning models with convolutional neural networks (CNNs) using the PyTorch open-source deep learning library in the Python programming language (version 3.8.5) [36]. The 80/20 data partitions into training/test sets for the GBM models (see above) were also used for these CNNs. Each segmented profile was interpolated to 64 × 64 pixels and normalized by computing the channel-level z-score per image. For both astrocyte and microglia CNNs, the model architecture was similar. Briefly, we implemented four convolutional layers, each followed by the rectified linear activation unit (ReLU). The first three convolutional layers were each followed by max pooling to create a down-sampled feature map, and then by a dropout layer for regularization. After the fourth convolutional layer, the feature vector was collapsed and passed to a fully connected neural network with three hidden layers. Finally, the *softmax* normalized exponential function was used to determine the classification probabilities.

*Hyperparameter optimization* The cross-entropy loss function was used. Other model hyperparameters—including the number of input and output channels, probabilities of each dropout layer, choice of optimizer (between Adam [37], stochastic gradient descent [SGD], and root mean square propagation [RMSprop], learning rate, and weight decay value (to enable L2 regularization and counteract overfitting)—were optimized via the Optuna hyperparameter tuning framework [38] using the multivariate tree-structured Parzen estimator algorithm [39]. The Optuna optimizer maximized the out-of-sample area under the receiver operating characteristic (ROC) curve (AUC), which in turn was determined by 3-fold cross-validation using the *scikit-learn* cross-validator within the 80% training set for each Optuna trial (i.e., Bayesian meta-optimization) [40]. Early stopping was applied within each fold if the validation loss failed to decrease after 10 epochs [41]. After cross-validation, each model was retrained on the full training data set (within each Optuna trial). The astrocyte and microglia CNNs were both optimized for 100 or more trials, after which the best performing model (based on average cross-validation AUC) was selected. Models were trained on a GPU workstation with two NVIDIA Quadro RTX 8000 graphics cards.

*Model performance* After model training, model performance was evaluated on the hold-out test set at the accuracy-maximizing thresholds and 95% confidence intervals were estimated by bootstrapping across 500 iterations. Both accuracy and AUC were computed as the primary outcomes reflecting the discriminatory power of the CNN between CTRL and AD astrocytes and microglia. Model performance was visualized via ROC curves, while the test set classification probabilities were visualized via histograms.

*Model interpretability* To investigate how the CNN model makes its classification decision—namely, whether this decision could be ascribed to pixels in specific subcellular locations and/or specific markers of each astrocyte or microglia image—we applied attribution functions of the Captum library for model interpretability in PyTorch [42]. Specifically, saliency maps [43], integrated gradients [44], and gradient-weighted class activation mappings (Grad-CAMs) based on the fourth convolutional layer [45] were computed and examined for astrocytes and microglia with high classification probabilities of either CTRL or AD.

### Interrogation of public single-nuclei RNA-seq-derived astrocytic and microglial subclusters

To determine whether the markers selected for our cyclic multiplex fluorescent immunohistochemistry are relevant to discern between homeostatic and reactive states of astrocytes and microglia, we interrogated the astrocytic and microglial subclusters from three published snRNA-seq studies on human AD and CTRL brains [8, 9, 12]. Mathys et al. [8] data were provided by the authors, Grubman et al. [9] data were obtained from adsn.ddnetbio.com, and Leng et al. [12] data were downloaded from www.synapse.org (syn21788402). The *Seurat* R package was used for all snRNA-seq analyses. These consisted of the following steps: (1) creation of Seurat objects: Seurat objects from Mathys et al. and Grubman et al. data sets were generated based on the counts, UMAP coordinates, and cell metadata, while the SingleCellExperiment objects provided by Leng et al. were converted to Seurat objects; (2) data normalization: for Mathys et al. and Grubman et al. data sets, we obtained the log-transformed expression measurements normalized by the total expression of each cell and scaled to 1 × 10^4^, whereas Leng et al. data were already normalized; and (3) bubble plot generation: finally, we generated bubble plots illustrating the relative expression of genes of interest (z-scores) across astrocytic and microglial subclusters.

### Statistical analyses

To determine whether individual astrocytic and microglial markers differ in signal intensity between CTRL and AD or across the phenotypic clusters obtained from the spectral clustering, we compared the MGI *z*-scores for each marker across groups. Because MGI data from astrocytes and microglia belonging to the same individual are not independent observations, we applied mixed effects regression models with diagnosis (CTRL vs. AD) or state (homeostatic vs. intermediate vs. reactive) as fixed effect, respectively, and subject ID as random effect in both cases, using the restricted maximum likelihood (REML) method in the *lmer* package in R [46]. The *p*-values of the pairwise comparisons were obtained using the Satterthwaite approximation with CTRL and homeostatic state as reference levels, respectively [46]. Differences in proportions of the astrocytes and microglia phenotypes (resulting from the spectral clustering) between CTRL and AD were tested with Chi-square (*χ*^2^) test for trend in GraphPad Prism version 9.1.0 (216) (GraphPad Software, La Jolla, CA). Significance level was set at a *p*-value < 0.05.

## Results

### Development of a cyclic multiplex fluorescent immunohistochemistry protocol for glial phenotyping

We developed a cyclic multiplex fluorescent immunohistochemistry protocol that allows the staining of the same FFPE section with up to 16 “off-the-shelf” primary antibodies distributed across 8 cycles of immunohistochemistry (Fig. 1a). Antibodies were selected to represent commonly used markers of homeostatic (astrocytes: ALDH1L1, EAAT1, EAAT2, GS; microglia: IBA1 and TMEM119) and reactive (astrocytes: GFAP, TSPO, VIM, YKL-40; microglia: CD68, FTL, MHC2, TSPO) glia [7, 47], as well as neurons (HuC/D), Aβ plaques, and NFTs (PHF1). Moreover, we confirmed that these combinations of astrocytic and microglial genes helped discriminate across astrocytic and microglial subclusters in published snRNA-seq studies on human AD and CTRL brains [8, 9, 12] (Fig. S3: Additional file 2). In each cycle, the microwaving of the sections in citrate buffer denatures and strips a large portion of both secondary and primary antibodies, while largely preserving or even retrieving some antigens [48]. The treatment of the sections with an oxidizing alkaline solution is known to quench the fluorescence emitted by any remaining fluorophore-conjugated secondary antibodies [21–23]. The sequence of primary antibodies was carefully determined by pilot studies and held constant for CTRL and AD sections: first, cross-reactivity of each cycle’s secondary antibodies with the primary antibodies used in the prior cycle was monitored by alternating the host species of the primary antibody and the target cell type (e.g., using a rabbit primary antibody for astrocytes and a mouse primary antibody for microglia in one cycle followed by a mouse primary antibody for astrocytes and a rabbit primary antibody for microglia in the next cycle); second, we attenuated cross-reactivity by ordering the sequence of cyclic immunohistochemistry with easier-to-strip antibodies in the first cycles (e.g., TSPO, TMEM119, MHC2, and CD68) and harder-to-strip antibodies in the later cycles (IBA1, GS, and Aβ and phospho-tau in AD samples). To facilitate the alignment of scanned images across cycles, we decided to immunostain for GFAP in every cycle because the repeated DAPI staining tended to become fainter after several cycles of immunohistochemistry, while GFAP immunohistochemistry is known to benefit from antigen retrieval with microwaving [48].

### Cyclic multiplex fluorescent immunohistochemistry followed by quantitative imaging analysis reveals complex phenotypic changes in Alzheimer’s disease astrocytes and microglia at single-cell resolution

Our cell profile segmentation workflow (see Methods and Fig. 1b) on the temporal neocortex of *n* = 7 CTRL and *n* = 7 AD subjects rendered a total of 5172 ALDH1L1+ astrocytes (CTRL: 320 [194–552]; AD: 355 [258–747], median [range]) and 6226 IBA1+ microglial profiles (CTRL: 407 [227–716]; AD: 484 [146–663], median [range]), which were included in subsequent analyses. The mean gray intensity (MGI) values for all other cell type-specific markers (i.e., EAAT1, EAAT2, GFAP, GS, TSPO, VIM, and YKL-40 for astrocytes, and CD68, FTL, MHC2, TMEM119, and TSPO for microglia) were measured and normalized. TSPO signal was measured in both ALDH1L1+ astrocytes and IBA1+ microglia because it is expressed by both glial cell types [49].

**Fig. 2:**
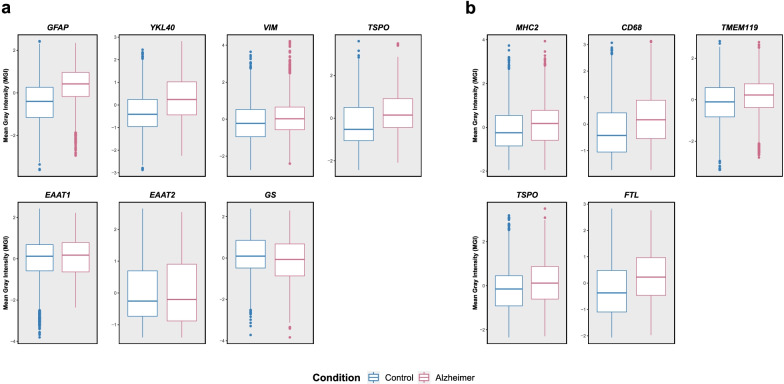
Quantitative characterization of astrocytes and microglia in control (CTRL) vs. Alzheimer's disease (AD) brains. Box and whisker plots depict the distribution (box: median and interquartile range [IQR]; whiskers: 1.5 × IQR) of mean gray intensity (MGI) *z*-scores for (**a**) each astrocytic marker and (**b**) each microglial marker across the CTRL and AD groups. Statistical comparisons between diagnostic groups are available in Table S3: Additional file 1

Box and whisker plots in Fig. 2a, b show the distribution of MGI for CTRL vs. AD subjects for astrocytes and microglia, respectively. Compared to CTRL astrocytes, AD astrocytes had higher levels of the reactive markers GFAP, YKL-40, and TSPO, slightly higher VIM and lower GS levels, and similar EAAT1 and EAAT2 levels (Fig. 2a). Mixed effects regression models controlling for correlation within subjects confirmed statistically significant differences between CTRL and AD astrocytes for GFAP (*p* = 0.025) and YKL-40 (*p* = 0.027), whereas the differences for EAAT1, EAAT2, GS, and VIM were not statistically significant (see Table S3: Additional file 1). Inspection of possible layer-specific effects revealed that the increase in GFAP and YKL-40 levels in AD vs. CTRL astrocytes was evident throughout all cortical layers analyzed (II through VI), whereas VIM and TSPO were clearly increased only in AD astrocytes from layers V and VI, GS was clearly reduced only in AD astrocytes from layers II to IV, and EAAT1 and EAAT2 exhibited a mixed trend depending on the cortical layer (see Fig. S4a: Additional file 2).

Similarly, compared to CTRL microglia, AD microglia appeared to exhibit higher levels of CD68, FTL, and TSPO, and slightly higher levels of MHC2 and TMEM119 (Fig. 2b); however, mixed effects regression models controlling for correlation within subjects only approached statistical significance for CD68 (*p* = 0.117) (see Table S3: Additional file 1). Box and whisker plots to visualize changes within AD microglia across cortical layers showed trends towards an increase in MHC2 and CD68 levels in layers III to V; TSPO in layers II and V; TMEM119 in layer III and, to a lesser extent, IV, V, and VI; and FTL in layers II, III, V, and VI (see Fig. S4b, Additional file 2).

### Spectral clustering of signal intensity data from thousands of single-cell high-plex images unveils three distinct astrocyte and microglia phenotypes across the normal aging to AD continuum

**Fig. 3:**
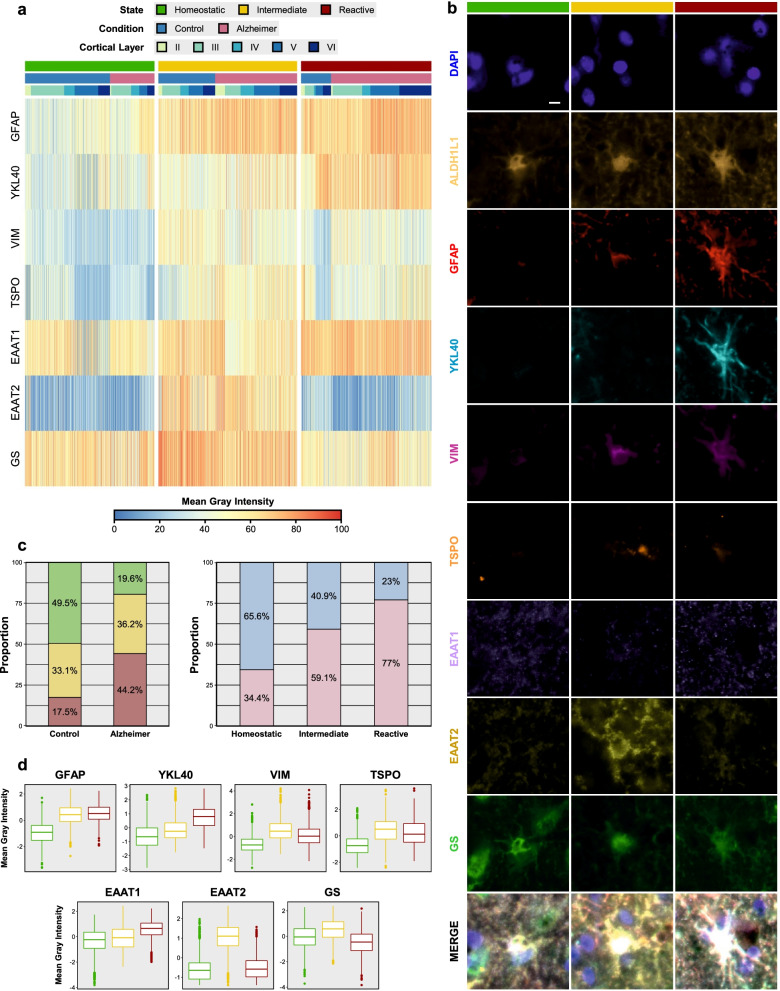
Unsupervised spectral clustering of astrocyte profiles reveals three distinct phenotypes. **a** Heatmap depicts the unsupervised spectral clustering of 5172 ALDH1L1+ astrocyte cell bodies based on their mean gray intensity (MGI) for the other 7 astrocytic markers. Note that MGI z-scores were scaled from 0 to 100 to facilitate comparison across markers. Three clusters are evident, which we termed “homeostatic,” “intermediate,” and “reactive.” **b** High-plex images from representative astrocytes of each of these clusters. Scale bar: 5 µm. **c** Stacked bar graphs show the proportions of astrocytic states by diagnosis and the proportions of diagnoses by astrocytic state. **d** Box and whisker plots illustrate the distribution (box: median and interquartile range [IQR]; whiskers: 1.5 × IQR) of MGI z-scores for each astrocytic marker across the homeostatic, intermediate, and reactive phenotypes. Statistical comparisons between states are available in Table S3, Additional file 1

**Fig. 4:**
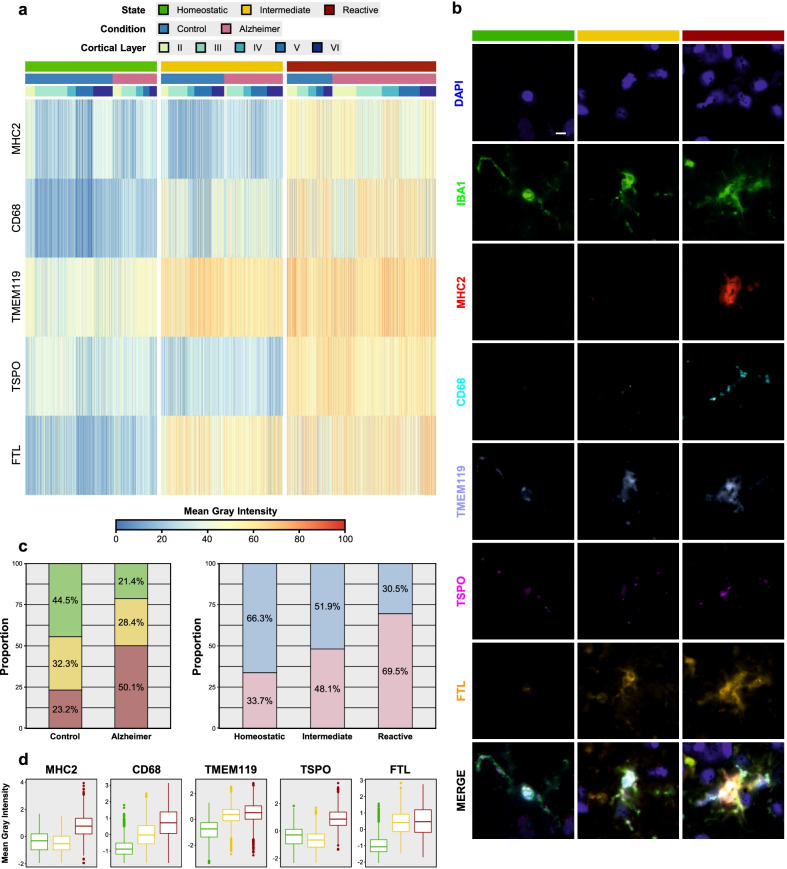
Unsupervised spectral clustering of microglial profiles reveals three distinct phenotypes. **a** Heatmap depicts the unsupervised spectral clustering of 6226 IBA1+ microglial profiles based on their mean gray intensity (MGI) for the other 5 microglial markers. Note that MGI z-scores were scaled from 0 to 100 to facilitate comparison across markers. Three clusters are evident, which we termed “homeostatic,” “intermediate,” and “reactive.” **b** High-plex images from representative microglial cells of each of these clusters. Scale bar: 5 µm. **c** Stacked bar graphs show the proportions of microglial states by diagnosis and the proportions of diagnoses by microglial state. **d** Box and whisker plots illustrate the distribution (box: median and interquartile range [IQR]; whiskers: 1.5 × IQR) of MGI *z*-scores for each microglial marker across the homeostatic, intermediate, and reactive phenotypes. Statistical comparisons between states are available in Table S3: Additional file 1

We next asked whether the combinations of these astrocytic and microglial markers allow the identification of more than two glial phenotypes. To this end, we performed spectral clustering on the log-normalized MGI *z*-scores. Spectral clustering is a graph-based, unsupervised machine learning technique that can outperform other clustering methods- [30]. Spectral clustering identified three distinct clusters of astrocytes and microglia, which we termed “homeostatic,” “intermediate,” and “reactive” (Figs. 3a and 4a). Examples of these distinct phenotypes or states of astrocytes and microglia can be found in Figs. 3b and 4b, respectively (see also Movie S1: Additional file 3 and Movie S2: Additional file 4).

Of note, homeostatic and reactive astrocytes were clearly predominant in CTRL and AD subjects, respectively, whereas intermediate astrocytes were more evenly distributed across CTRL and AD individuals (see annotation bar in Fig. 3a heatmap). Specifically, 49.5% of CTRL astrocytes were classified as homeostatic, 33.1% as intermediate, and 17.5% as reactive, whereas the reverse (19.6%, 36.2%, and 44.2%) was true for AD astrocytes. In other words, 65.6% of all homeostatic astrocytes belonged to CTRL subjects and 77.0% of all reactive astrocytes belonged to AD individuals, whereas 59.1% of intermediate astrocytes corresponded to AD and 40.9% to CTRL subjects (Fig. 3c). These phenotypic differences between CTRL and AD groups were statistically very significant (*χ*^2^ for trend = 624.2, *df* = 1, *p* < 0.0001). Compared to their homeostatic counterparts, reactive astrocytes were characterized by a substantial increase in GFAP and YKL-40, a modest elevation of VIM and TSPO as well as EAAT1, and reduced expression of GS. Intermediate astrocytes were defined by a marked increase in EAAT2 and a modest increase in GS, with GFAP and YKL-40 levels between those of homeostatic and reactive astrocytes (Fig. 3d). Mixed effects models controlling for correlation within subjects and with homeostatic state as reference revealed that these differences between homeostatic vs. intermediate and reactive phenotypes were also statistically highly significant (Table S3: Additional file 1). No clear layer-specific effect was evident in these trends for any of the astrocyte markers by visual inspection of the data (see box and whisker plots in Fig. S5a: Additional file 2 and cortical layer annotation bar above heatmap in Fig. 3a).

Similarly, homeostatic was the predominant phenotype among CTRL microglia (44.5%), followed by intermediate (32.3%) and reactive (23.2%), whereas reactive microglia represented the majority among AD microglia (50.1%), followed by intermediate (28.4%) and homeostatic (21.5%). In other words, 66.3% of homeostatic microglia belonged to CTRL subjects and 69.5% of reactive microglia to AD subjects, whereas 51.9% of intermediate microglia corresponded to CTRL subjects and 48.1% to AD individuals (Fig. 4c). Again, these phenotypic differences between CTRL and AD groups were statistically very significant (*χ*^2^ for trend = 559.9, *df* = 1, *p* < 0.0001). Compared to homeostatic microglia, reactive microglia showed increased expression of all markers, including surprisingly the purported homeostatic marker TMEM119, whereas intermediate microglia exhibited increased FTL and TMEM119 as well as intermediate levels of CD68 (Fig. 4d). Mixed effects models controlling for within-subject correlations and with homeostatic state as reference revealed highly significant increases for MHC2, CD68, TMEM119, and FTL for both intermediate and reactive phenotypes, whereas TSPO was only statistically significantly increased in reactive vs. homeostatic, but not in intermediate vs. homeostatic (*p* = 0.483) (Table S3: Additional file 1). Again, the marker profiles defining homeostatic, intermediate, and reactive microglia were essentially applicable in all cortical layers (II through VI), with no evident layer-specific differences (see Fig. S5b: Additional File 2 and cortical layer annotation bar above spectral clustering heatmap in Fig. 4a).

### Effect of proximity to AD neuropathological changes on astrocyte and microglia phenotypes

**Fig. 5:**
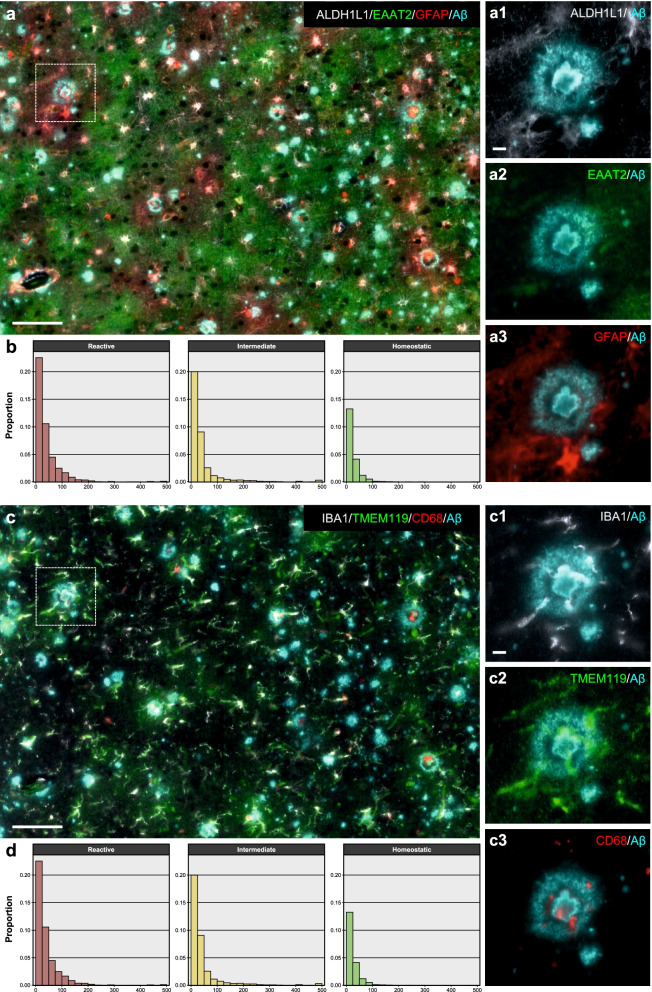
Effect of proximity to AD neuropathological changes (Aβ plaques or PHF1+ NFTs) on astrocytic and microglial phenotypes from AD subjects. **a** Representative high-plex image of astrocytes from an AD subject. For clarity, only ALDH1L1, EAAT2, and GFAP markers are shown together with Aβ. Scale bar: 100 µm, insets a1–a3: 10 µm. **b** Histograms show the proportion of each astrocytic phenotype with respect to all AD astrocytes as a function of the distance (µm, *x* axis) to the nearest Aβ plaque or PHF1+ NFT. Reactive astrocytes were relatively more closely associated with AD neuropathological changes than intermediate astrocytes, and these more than homeostatic astrocytes. **c** Representative high-plex image of microglia from the same field of the same AD subject. For clarity, only IBA1, TMEM119, and CD68 markers are shown together with Aβ. Scale bar: 100 µm, insets c1–c3: 10 µm. **d** Histograms indicate the proportion of each microglial phenotype with respect to all AD microglial profiles as a function of the distance (µm, *x* axis) to the nearest Aβ plaque or PHF1+ NFT. Reactive microglia were relatively more closely associated with AD neuropathological changes than intermediate microglia, and these more than homeostatic microglia

AD neuropathological changes, especially Aβ plaques but also NFTs, are thought to trigger astrocytic and microglial reactions [2–5]. Thus, we asked whether the above three phenotypes differed by their proximity to Aβ plaques and PHF1+ NFTs. Histograms in Fig. 5b, d show the frequency distribution of each of the three astrocyte and microglia phenotypes in AD subjects with respect to the total number of each cell type profiles as a function of distance to the nearest Aβ plaque or PHF1+ NFT in 25 μm intervals. Indeed, an effect of proximity to AD pathology was noted with relatively higher proportions of reactive astrocytes and microglia located within 50 μm of an AD lesion. Since two of the seven CTRL subjects had abundant Aβ plaques in their temporal neocortex, we asked whether an effect of proximity to Aβ plaques was already discernible in these cases. We observed that, although reactive astrocytes and microglia tended to reside within 50 μm of an Aβ plaque, significant proportions of homeostatic astrocytes and microglia were also located within that plaque boundary, suggesting less toxicity of Aβ plaques in CTRL vs. AD brains (Fig. S6: Additional file 2). Indeed, plaques from these two CTRL subjects had less neuritic changes in the PHF1 immunostaining (Fig. S7: Additional file 2).

### Gradient boosting machine models on signal intensity data from thousands of single-cell high-plex images accurately predict AD diagnosis

**Fig. 6:**
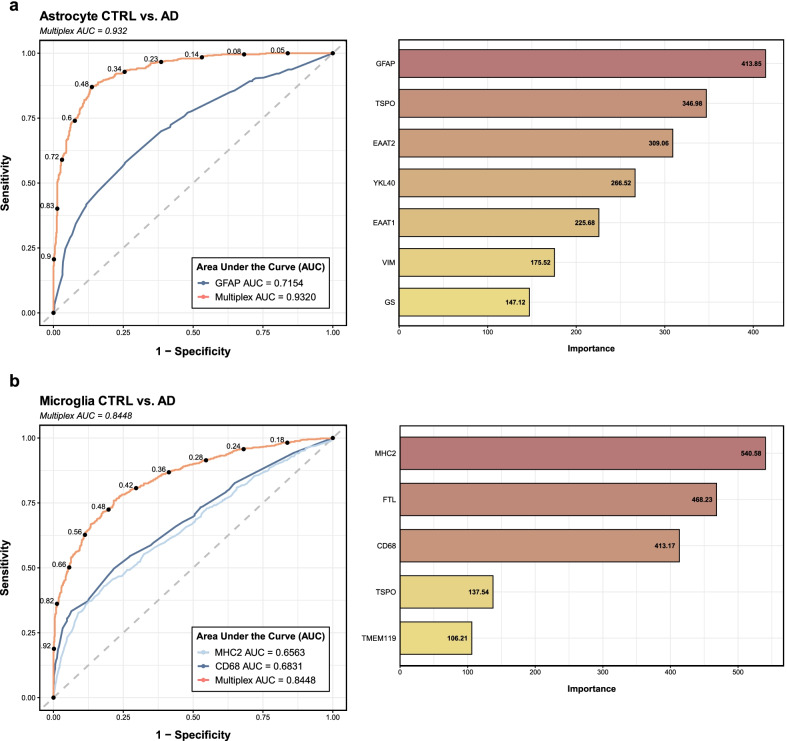
Gradient boosting machine models accurately discriminate CTRL vs. AD astrocytes and microglia. Receiver operating characteristic (ROC) curves demonstrate the high discriminative power of the gradient boosting machine (GBM) models to discern between CTRL and AD (**a**) astrocytes and (**b**) microglia based on mean gray intensity (MGI) data from thousands of high-plex single-cell profiles. Compare with the performance of GBM models trained on single marker intensity data, namely (**a**) GFAP and (**b**) MHC2 or CD68. Rankings of the variable importance scores shown in the horizontal bar plots reveal the most relevant markers for each classification task, respectively

Next, we aimed to develop a supervised machine learning algorithm to predict the diagnosis of CTRL vs. AD based on the intensity profile of high-plex astrocyte or microglia profiles with the hypothesis that the phenotypic characterization of these glial cells could have diagnostic value. To this end, we trained stochastic gradient boosting machine (GBM) models on the normalized MGI values of either astrocytic or microglial markers. GBM is a supervised machine learning method that uses decision trees to perform classification tasks (see Methods and Table S4: Additional file 1). The astrocyte GBM model performed with an accuracy of 86.57% (95% CI [84.54–88.60]) and an AUC of 0.9320 (95% CI [0.9157–0.9462]), with a *p*-value of accuracy better than the no-information rate (ACC > NIR) of < 2e−16 (Fig. 6a). Similarly, the microglia GBM model performed with an accuracy of 77.19% (95% CI [75.46–79.84]) and an AUC of 0.8448 (95% CI [0.8233–0.8666], with a *p*-value (ACC > NIR) < 2.2e−16 (Fig. 6b). Notably, both models outperformed GBM algorithms trained using only MGI values from one of the classic markers of reactive astrocytes (GFAP: accuracy 67.25%, AUC = 0.7154) or microglia (MHC2: accuracy 61.93%, AUC = 0.6563; CD68: accuracy 63.61%, AUC = 0.6831), demonstrating that a combination of reactive and homeostatic markers adds predictive value for the CTRL vs. AD classification.

We also sought to determine the relative contribution of each marker to the classification of astrocytes and microglia as CTRL vs. AD. Interestingly, the relative variable importance scores indicated that the reactive markers GFAP and TSPO were most determinant for the CTRL vs. AD classification of astrocytes, followed by EAAT2, YKL-40, EAAT1, VIM, and GS (Fig. 6a). Likewise, the classifier ranked MHC2 as the most important marker for the CTRL vs. AD classification of microglia, followed by FTL, CD68, TSPO, and TMEM119 (Fig. 6b). Thus, of all markers analyzed, the classic reactive markers GFAP and MHC2 (rather than CD68) are the most predictive to discriminate CTRL vs. AD astrocytes and microglia, respectively.

Furthermore, we asked whether the supervised GBM models could also predict the phenotypic classification rendered by the unsupervised spectral clustering. To this end, we constructed additional GBM models on the normalized MGI *z*-scores for both astrocytes and microglia using the classification labels “homeostatic,” “intermediate,” and “reactive” assigned by spectral clustering. The astrocyte GBM model had an accuracy of 96.52% (AUC = 0.9983, *p*-value [Acc > NIR] < 2e−16) to classify homeostatic, intermediate, and reactive astrocytes (Fig. S8a: Additional file 2), whereas the microglia GBM had an accuracy of 96.79% (AUC = 0.9985, *p*-value [Acc > NIR] < 2e−16) to classify across the same microglial states (Fig. S8b: Additional file 2), suggesting that the astrocytic and microglial markers can accurately discriminate between the three states. Remarkably, the ranking of variable importance scores indicated that the marker EAAT2 was the most influential marker for this phenotypic classification, followed by the reactive markers GFAP and YKL-40 and, with much lower importance, GS, TSPO, VIM, and EAAT1 (Fig. S8a: Additional file 2). Regarding microglia, TSPO and FTL were most determinant for their classification in homeostatic, intermediate, or reactive, followed by MHC2, TMEM119, and CD68 (Fig. S8b: Additional file 2).

### Convolutional neural networks accurately classify CTRL and AD astrocytes and microglia

**Fig. 7:**
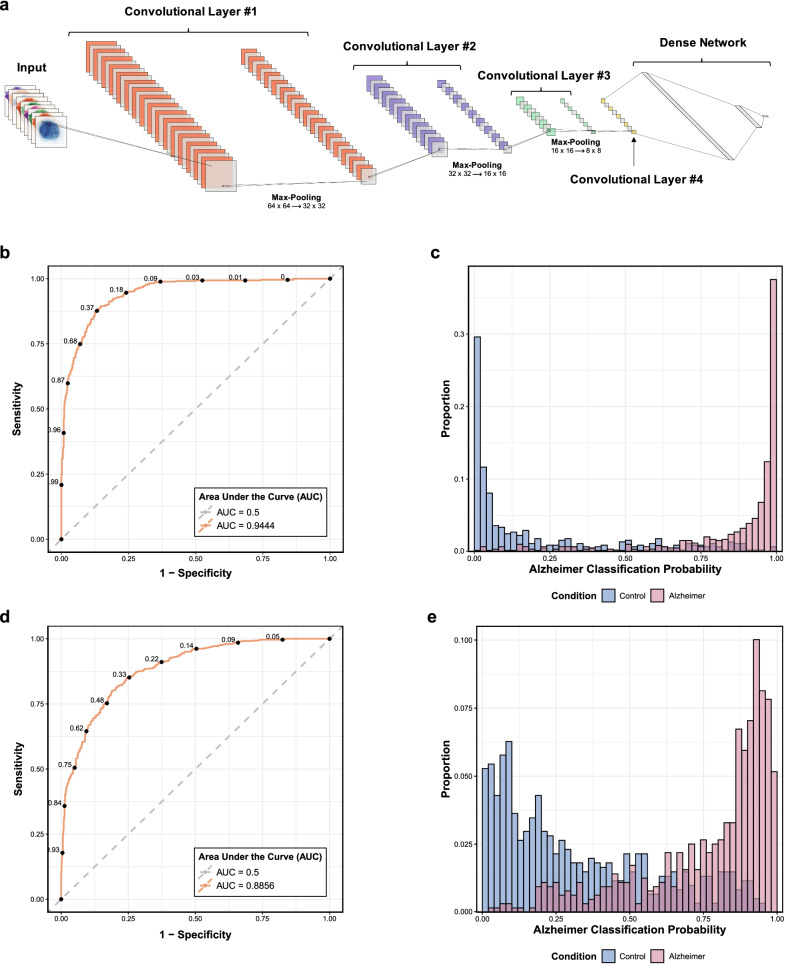
Deep learning with convolutional neural networks accurately predicts CTRL vs. AD astrocytes and microglia. **a** Architecture of the convolutional neural networks (CNNs) used for deep learning of image features from astrocyte and microglial profiles (see details in Methods section). Receiver operating characteristic (ROC) curves demonstrate the performance of the CNN model to predict the diagnosis of CTRL vs. AD based on the features of (**b**) astrocytic or (**d**) microglial high-plex images. Histograms show the within-group proportions of (**c**) astrocytes or (**e**) microglia as a function of the AD classification probability stratified by their true label (i.e., CTRL or AD). Note that both CTRL astrocytes and CTRL microglia (blue bars) tend to have a probability of AD diagnosis closer to zero, whereas AD glia (red bars) tend to be correctly classified as AD

Finally, we sought to test whether a deep learning classifier could effectively distinguish between CTRL and AD astrocytes or microglia based on all image features (e.g., pixel-level subcellular location, cell morphology, etc.) rather than solely on MGI. To this end, we created convolutional neural networks (CNNs) to classify astrocytes and microglia as CTRL vs. AD. The model architecture is shown in Fig. 7a and the optimal hyperparameters determined via Bayesian optimization are reported in Table S5, Additional file 1. In both cases, the most important hyperparameter to tune with respect to the objective value (i.e., the cross-validation AUC) was the learning rate. The astrocyte CNN performed on the hold-out test set of 1035 astrocytic profiles with an accuracy of 87.44% (95% CI [85.65–89.47]) and an AUC of 0.9444 (95% CI [0.9318–0.9561]) (Fig. 7b and Table S4: Additional file 1). Meanwhile, the microglia CNN performed on the hold-out test set of 1245 microglial profiles with an accuracy of 80.56% (95% CI [78.47–83.05]) and an AUC of 0.8856 (95% CI [0.8684–0.9032]) (Fig. 7c and Table S4: Additional file 1). To facilitate CNN model interpretability, examples of saliency, integrated gradient, and GradCAM pixel maps are shown in Figs. S9 and S10: Additional file 2 for correctly classified astrocytes and microglia, respectively. Taken together with the GBM classifier, this deep learning approach supports the idea that profiling the complexity of glial reactions discerns CTRL from AD brains with high accuracy.

## Discussion

We have developed a novel protocol of cyclic multiplex fluorescent immunohistochemistry on human postmortem FFPE sections followed by single-cell segmentation and machine learning-based classification to better characterize glial responses in AD. Our methodology consists of an iterative 3-plex fluorescent immunohistochemistry with primary antibodies and fluorophore-conjugated secondary antibodies in each cycle. After every cycle, sections are scanned, their coverslips are carefully removed with little or no damage to the tissue, and two procedures incorporated from other methods are performed: an antibody denaturation/stripping step by microwaving sections in citrate buffer as in the Opal method [20], and a fluorescence quenching step with an alkaline oxidizing solution as in the tissue-based cyclic immunofluorescence (t-CyCIF) method [21–23]. Since most primary antibodies used here are not commercially available in directly fluorophore-conjugated versions, and fluorophore labeling kits are expensive and may cause non-specific staining artifacts, we decided to use commercial unconjugated primary antibodies followed by fluorophore-conjugated secondary antibodies. Because most reliable primary antibodies are made in mouse or rabbit, careful testing and ordering of the sequence of immunohistochemistry cycles was required to detect and prevent cross-species reactivity of the secondary antibodies. We found that some primary antibodies are easier to strip from the FFPE sections (e.g., CD68, MHC2, and TMEM119) than others (e.g., Aβ and PHF1), and that alternating the host species of primary antibodies for microglia vs. astrocytes in consecutive cycles minimized the chances of cross-species reactivity; therefore, both factors were considered in the final sequence of immunostainings.

Selected antibodies included a combination of homeostatic and reactive markers. For astrocytes, we selected the glutamate transporters EAAT1 and EAAT2 as well as GS as homeostatic markers, because one of the main functions of cortical protoplasmic astrocytes is the uptake and recycling of the glutamate released by the excitatory neurons to the synaptic cleft. Conversely, GFAP, VIM, and YKL-40 are well-established markers of reactive astrocytes [6, 50]. For microglia, CD68 and MHC2 are widely used as reactive microglia markers indicative of phagocytosis and antigen presentation [2–5], respectively. TMEM119 was first described as a homeostatic microglial marker in wild-type mice [51], and one of the most downregulated genes in disease-associated microglia (DAM) in 5xFAD transgenic mice [52]. Ferritin is an iron-binding protein involved in iron homeostasis, although some researchers have linked it with a dystrophic and/or senescent microglial phenotype that is increased in AD [53]. TSPO is expressed by both microglia and astrocytes and is a target for PET radioligands of reactive glia [49].

Spectral clustering of signal intensity data from the combination of available markers discriminated at least three states in both glial cell types, suggesting that the qualifiers “homeostatic” and “reactive” alone may be insufficient to describe the heterogeneity of these glial cells in the healthy and AD brains [50]. Compared to the homeostatic state, the reactive astrocyte phenotype had expectedly high levels of all the aforementioned reactive markers but also lower levels of the homeostatic marker GS. By contrast, the intermediate astrocyte phenotype had intermediate (YKL-40) or high (GFAP, TSPO, VIM) levels of reactive markers and remarkably high levels of EAAT2. *SLC1A2* and *GLUL* transcripts, encoding for EAAT2 and GS, respectively, are downregulated in AD astrocytes in snRNA-seq studies [8–10, 12] (Fig. S3: Additional file 2); however, immunohistochemical [54] and biochemical [55] studies have yielded conflicting results. Conversely, EAAT1 levels are thought to be preserved [54, 55]. Hence, the high EAAT2 and GS expression in intermediate astrocytes could represent a mechanism of resilience to the neuronal glutamate excitotoxicity induced by the presence of AD neuropathological changes, as has been described in the entorhinal cortex [56].

Similarly, relative to the homeostatic state, the reactive microglial phenotype exhibited high levels of all reactive markers and, surprisingly, also of the homeostatic marker TMEM119, whereas the intermediate microglia phenotype had intermediate (CD68) or low (MHC2, TSPO) levels of reactive markers, and intermediate levels of TMEM119. These findings are consistent with a snRNA-seq study reporting an upregulation of the *TMEM119* transcript in human AD microglia [10] and contrary to the downregulation in DAM reported in mouse models of cerebral β-amyloidosis [52]. Finally, the high levels of FTL in the intermediate phenotype suggests a compensatory mechanism against iron accumulation and subsequent generation of toxic reactive oxygen species in microglia [57], analogous to the EAAT2 upregulation in intermediate astrocytes.

Glial reactions initially occur within the vicinity of Aβ plaques, which is thought to be a toxic microenvironment. Indeed, our prior stereology-based spatial quantitative postmortem studies with a single marker demonstrated an accumulation of GFAP+ astrocytes as well as CD68+ and MHC2+ microglia within 50 μm from both the plaque edge and, to a lesser extent, NFTs [2–5], which seems to track with disease progression [2, 4, 5, 54]. In the present study, the more complex reactive phenotypes described here with multiple markers also predominated in the proximity of Aβ plaques and NFTs in AD subjects, whereas in the two CTRL subjects with abundant Aβ plaques many of the astrocytes and microglial cells within that boundary were still homeostatic. This finding was likely related to the lesser neuritic component of their plaques but warrants further confirmation in brains of individuals resilient to high levels of AD pathology (so-called high-pathology controls, mismatch AD, or asymptomatic AD) [56, 58, 59]. While various spatial -omics methods are currently attempting to link complex cellular phenotypes with pathological features of brain tissue, our immunohistochemistry-based approach was particularly adept at this task thanks to its single-cell resolution, allowing us to better understand the relationship between the pathognomonic lesions of AD and these cellular changes in astrocytes and microglia.

Prior studies have applied various machine learning methods to segment and quantify IBA1+ microglia [60–64], classify IBA1+ microglia morphological subtypes [65], and segment and quantify GFAP+ or ALDH1L1+ astrocytes [61, 66, 67] from mouse or human brain sections. Here, we built upon these methods by taking advantage of our large data set of 8-plex astrocyte and 6-plex microglia images to investigate whether machine learning algorithms can accurately predict the diagnosis of CTRL vs. AD and the classification in three phenotypes or states developed with unsupervised clustering of the signal intensity data. Indeed, GBM classifiers applied on the same signal intensity data were highly accurate at classifying CTRL vs. AD glia and, based on the expression levels of the markers used here, lent additional support to the existence of three distinct phenotypes of astrocytes and microglia. Interestingly, these models also determined that the classic reactive markers GFAP and MHC2 are the most influential in the CTRL vs. AD classification of astrocytes and microglia, respectively, whereas EAAT2 and TSPO are the most important for their phenotypic classification. Finally, deep learning of high-plex images of thousands of individual glial profiles via CNNs also enabled a highly accurate classification of CTRL vs. AD membership for both astrocytes and microglia at the single-cell level, with AUCs of 0.94 and 0.89, respectively. Since CNN models learn all image features (rather than just mean pixel intensity) to infer a classification probability, our CNN classifiers provide an unbiased confirmation of the profound changes that astrocytes and microglia undergo in the AD brain. Furthermore, the high predictive power of the CNNs described here suggests that these deep learning models could be used to assign scores (i.e., classification probabilities) of disease association at the single-cell level, which could be combined across brain regions for staging of glial responses to AD neuropathology.

## Conclusions

In summary, cyclic multiplex fluorescent immunohistochemistry allows the thorough phenotyping of individual glial cells in postmortem brain specimens to identify distinct states in health and disease. Besides the homeostatic and reactive, we identified a novel “intermediate” state of both astrocytes and microglia, which may represent a resilience mechanism or, alternatively, a transitional state between homeostatic and reactive. We were also able to demonstrate the spatial relationships between the classic AD lesions and these glial phenotypes, an aspect of analysis that is difficult to achieve at this resolution with other methods. This and similar methods could help understand the heterogeneity of glial responses in AD and other neurodegenerative diseases, validate clusters derived from snRNA-seq and scRNA-seq studies while placing those changes into the appropriate spatial context, and inform ongoing biomarker discovery efforts.

## Supplementary Information

**Additional File 1: Table S1.** Demographic and neuropathological characteristics of study subjects. Description: Abbreviations: ADNC = AD neuropathological changes; APOE = Apolipoprotein E genotype; CAA = cerebral amyloid angiopathy; CVD = cerebrovascular disease; F = female; LBD = Lewy body disease; M = male; NA = Not available/applicable; NOS = not otherwise specified; NP Dx = neuropathological diagnosis. **Table S2.** Primary and secondary antibodies used in this study and sequence of immunohistochemistry cycles. Description: Note: GFAP and DAPI detection are needed in all the cycles to guarantee an adequate alignment of the images. Abbreviations: AF488 = AlexaFluor 488; Cy = cyanine; Dk = donkey; Gt = goat; Ms = mouse; Rb = rabbit. All secondary antibodies were purchased from Jackson ImmunoResearch Labs, West Grove, PA. **Table S3.** Results of mixed effects regression models. Description: Results of mixed effects regression models with diagnosis (CTRL vs. AD) or state (homeostatic vs. intermediate vs. reactive) as a fixed effect, respectively, and subject ID as random effect in both cases, are reported. **Table S4.** Model performance statistics for CTRL vs. AD binary classifiers. Description: Model performance statistics for the binary classification task of CTRL vs. AD for both the gradient boosting machine (GBM) and the convolutional neural network (CNN) machine learning models are reported. For all heuristics except for AUC and AUCPR (which are not threshold-dependent), the threshold was chosen by maximizing the accuracy. 95% confidence intervals were estimated by bootstrapping the hold-out test set across 500 iterations. **Table S5.** Results of Bayesian hyperparameter optimization. Description: The final hyperparameters determined by the Optuna hyperparameter tuning framework are reported. The Optuna optimizer maximized the out-of-sample area under the receiver operating characteristic (ROC) curve (AUC), which in turn was determined by 3-fold cross-validation for each trial.

**Additional File 2: Figure S1.** A β pathology in the temporal pole cortex. Description: Immunohistochemistry for Aβ (mouse monoclonal antibody, clone 6F/3D, Agilent, #M0872, 1:600) with peroxidase/DAB was performed in nearly-adjacent sections to those used for cyclic multiplex fluorescent immunohistochemistry in a Leica BOND-III automated stainer. Sections were counterstained with hematoxylin. Scale bars: 5 mm, insets 200 μm. **Figure S2.** Phospho-tau pathology in the temporal pole cortex. Description: Immunohistochemsitry for phospho-tau^Ser202/Thr205^(mouse monoclonal antibody, clone AT8, Thermo-Scientific, #MN1020, 1:10,000) with peroxidase/DAB was performed in nearly-adjacent sections to those used for cyclic multiplex fluorescent immunohistochemistry in a Leica BOND-III automated stainer. Sections were counterstained with hematoxylin. Scale bars: 5 mm, insets 200 μm. **Figure S3.** Expression levels of selected markers across astrocytic and microglial subclusters from public single-nuclei RNA-seq studies. Description: Bubble plots illustrate the percent of nuclei (bubble size) and the gene expression levels (z-scores, color bar) of the astrocytic and microglial markers used in our cyclic multiplex fluorescent immunohistochemistry protocol across the astrocytic and microglial subclusters rendered by several published single-nuclei RNA-seq data sets. Note that our set of markers discriminates some of these transcriptomic subclusters. **Figure S4.** Characterization of astrocytes and microglia in AD vs. CTRL by cortical layer. Description: Box and whisker plots illustrate the distribution (box: median and interquartile range [IQR]; whiskers: 1.5 × IQR) of mean gray intensity (MGI) z-scores for (a) each astrocytic marker and (b) each microglial marker across the CTRL and AD groups by cortical layer. Only layers II to VI were included in this study. **Figure S5.** Characterization of astrocytic and microglial states by cortical layer. Description: Box and whisker plots show the distribution (box: median and interquartile range [IQR]; whiskers: 1.5 × IQR) of mean gray intensity (MGI) z-scores for each astrocytic (a) or microglial (b) marker across the three phenotypes by cortical layer. Only layers II to VI were included in this study. **Figure S6.** Effects of proximity to AD neuropathological changes on astrocytic and microglial phenotypes from two CTRL subjects with abundant Aβ plaques. Description: (a) Representative high-plex image of astrocytes from a CTRL subject with abundant Aβ plaques; note the differences with AD astrocytes in Fig. 5a. For clarity, only ALDH1L1, EAAT2, and GFAP markers are shown together with Aβ. Scale bar: 100 µm, insets a1–a3: 10 µm. (b) Histograms show the proportion of each astrocyte phenotype in n=2 CTRL subjects with abundant Aβ plaques relative to all their astrocytes as a function of their distance (µm, ***x*** axis) to the nearest Aβ plaque. Note that there are equal numbers of astrocytes within 25 µm from the nearest Aβ plaque classified as homeostatic, intermediate, or reactive. (c) Representative high-plex image of microglia from the same field of the same CTRL with abundant Aβ plaques; note the differences when compared to AD microglia in Fig. 5c. For clarity, only IBA1, TMEM119, and CD68 markers are shown together with Aβ. Scale bar: 100 µm, insets c1–c3: 10 µm. (d) Histograms indicate the proportion of each microglial phenotype in n=2 CTRL subjects with abundant Aβ plaques relative to all their microglial profiles as a function of their distance (µm, ***x*** axis) to the nearest Aβ plaque. Note that most microglia in the vicinity of Aβ plaques were classified as homeostatic, suggesting that their phenotypic transition to intermediate and reactive had not yet occurred. **Figure S7.** Differences in neuritic component of Aβ plaques from CTRL and AD subjects. Description: Representative images of Aβ and phospho-tau (PHF1) immunohistochemistry corresponding to the same fields of the AD and CTRL subjects shown in Fig. 5 and Fig. S6, respectively. Note the differences in the PHF1+ neuritic changes between CTRL and AD Aβ plaques. Scale bar: 100 µm, insets a1 and b1: 10 µm. **Figure S8.** Gradient boosting machine models accurately discriminate between glial phenotypes. Description: Receiver operating characteristic (ROC) curves demonstrate the high discriminative power of the gradient boosting machine (GBM) models to discern between states (i.e., homeostatic vs. intermediate vs. reactive) of (a) astrocytes and (b) microglia based on mean gray intensity (MGI) data from thousands of high-plex single-cell profiles. Rankings of the variable importance scores shown in the horizontal bar plots reveal the most relevant markers for each classification task, respectively. **Figure S9.** Application of deep learning model interpretability functions to astrocytes with extreme classification probabilities. Description: Examples of the convolutional neural network (CNN) model interpretability functions applied to astrocytes with extreme classification probabilities (i.e., confident and correct predictions). Columns 1 and 5 show DAPI and all astrocyte markers of the high-plex image of a single astrocyte cell body from a CTRL and an AD subject, respectively, after performing the CNN normalization steps described (i.e., segmentation, interpolation, channel-level z-score). Hence, the signal intensity is represented by dynamic range rather than by pixel intensity. Columns 2–4 and 6–8 show the saliency (2 and 6), integrated gradient (3 and 7), and GradCAM (4 and 8) maps, which illustrate the pixels of each marker that the CNN considered most important for the classification of these two astrocytes as CTRL or AD. **Figure S10.** Application of deep learning model interpretability functions to microglia with extreme classification probabilities. Description: Examples of the convolutional neural network (CNN) model interpretability functions applied to microglia with extreme classification probabilities (i.e., confident and correct predictions). Columns 1 and 5 show DAPI and all microglial markers of the high-plex image of a single microglial cell from a CTRL and an AD subject, respectively, after performing the CNN normalization steps described (i.e., segmentation, interpolation, channel-level z-score). Hence, the signal intensity is represented by dynamic range rather than by pixel intensity. Columns 2–4 and 6–8 show the saliency (2 and 6), integrated gradient (3 and 7), and GradCAM (4 and 8) maps, which illustrate the pixels of each marker that the CNN considered most important for the classification of these two microglia as CTRL or AD.

**Additional File 3: Movie S1.** High-plex images of representative astrocytes from the “homeostatic,” “intermediate,” and “reactive” clusters. Description: Scale bar: 5 µm.

**Additional File 4: Movie S2.** High-plex images of representative microglia from the “homeostatic,” “intermediate,” and “reactive” clusters. Description: Scale bar: 5 µm.

## Data Availability

All data needed to evaluate the conclusions in the paper are present in the paper and/or additional files. This study did not generate new unique reagents. ImageJ, R, and Python code for all computational analyses is available on GitHub at https://github.com/serrano-pozo-lab/glia-ihc.

## Abbreviations

Aβ: Amyloid beta peptide
AD: Alzheimer’s disease
ALDH1L1: Aldehyde dehydrogenase 1 L1
AUC: Area under the curve
CD68: Cluster differentiation 68
CNN: Convolutional neural network
CTRL: Control
DAM: Disease-associated microglia
DAPI: 4′,6-Diamidino-2-phenylindole
EAAT1: Excitatory amino acid transporter 1
EAAT2: Excitatory amino acid transporter 2
FFPE: Formalin-fixed paraffin-embedded
GBM: Gradient boosting machine
FTL: Ferritin light chain
GFAP: Glial fibrillary acidic protein
GS: Glutamine synthetase
IBA1: Ionized calcium-binding adaptor molecule 1
MADRC: Massachusetts Alzheimer’s Disease Research Center
MGI: Mean gray intensity
MHC2: Major histocompatibility complex II
NDS: Normal donkey serum
NFTs: Neurofibrillary tangles
PHF1: Paired helical filament 1
ROC: Receiver operating characteristic
ROI: Region of interest
RT: Room temperature
scRNA-seq: Single-cell RNA sequencing
snRNA-seq: Single-nuclei RNA sequencing
TBS: Tris-buffered saline
t-CyCIF: Tissue-based cyclic immunofluorescence
TMEM119: Transmembrane protein 119
TSPO: Translocator protein 18 kDa
VIM: Vimentin

## References

[1] Serrano-Pozo A, Frosch MP, Masliah E, Hyman BT (2011). Neuropathological alterations in Alzheimer disease. Cold Spring Harb Perspect Med.

[2] Serrano-Pozo A, Mielke ML, Gómez-Isla T, Betensky RA, Growdon JH, Frosch MP (2011). Reactive glia not only associates with plaques but also parallels tangles in Alzheimer’s disease. Am J Pathol.

[3] Serrano-Pozo A, Muzikansky A, Gómez-Isla T, Growdon JH, Betensky RA, Frosch MP (2013). Differential relationships of reactive astrocytes and microglia to fibrillar amyloid deposits in Alzheimer disease. J Neuropathol Exp Neurol.

[4] Serrano-Pozo A, Gómez-Isla T, Growdon JH, Frosch MP, Hyman BT (2013). A phenotypic change but not proliferation underlies glial responses in Alzheimer disease. Am J Pathol.

[5] Serrano-Pozo A, Betensky RA, Frosch MP, Hyman BT (2016). Plaque-associated local toxicity increases over the clinical course of alzheimer disease. Am J Pathol.

[6] Perez-Nievas BG, Serrano-Pozo A (2018). Deciphering the astrocyte reaction in Alzheimer’s disease. Front Aging Neurosci.

[7] de Navas LV, Noori A, Merrill E, Das S, Hyman BT, Serrano-Pozo A (2021). Systematic review of human post-mortem immunohistochemical studies and bioinformatics analyses unveil the complexity of astrocyte reaction in Alzheimer’s disease. Neuropathol Appl Neurobiol.

[8] Mathys H, Davila-Velderrain J, Peng Z, Gao F, Mohammadi S, Young JZ (2019). Single-cell transcriptomic analysis of Alzheimer’s disease. Nature.

[9] Grubman A, Chew G, Ouyang JF, Sun G, Choo XY, McLean C (2019). A single-cell atlas of entorhinal cortex from individuals with Alzheimer’s disease reveals cell-type-specific gene expression regulation. Nat Neurosci.

[10] Zhou Y, Song WM, Andhey PS, Swain A, Levy T, Miller KR (2020). Human and mouse single-nucleus transcriptomics reveal TREM2-dependent and TREM2-independent cellular responses in Alzheimer’s disease. Nat Med.

[11] Olah M, Menon V, Habib N, Taga MF, Ma Y, Yung CJ (2020). Single cell RNA sequencing of human microglia uncovers a subset associated with Alzheimer’s disease. Nat Commun.

[12] Leng K, Li E, Eser R, Piergies A, Sit R, Tan M (2021). Molecular characterization of selectively vulnerable neurons in Alzheimer’s disease. Nat Neurosci.

[13] Lau S-F, Cao H, Fu AKY, Ip NY (2020). Single-nucleus transcriptome analysis reveals dysregulation of angiogenic endothelial cells and neuroprotective glia in Alzheimer’s disease. Proc Natl Acad Sci USA.

[14] Gerrits E, Brouwer N, Kooistra SM, Woodbury ME, Vermeiren Y, Lambourne M (2021). Distinct amyloid-β and tau-associated microglia profiles in Alzheimer’s disease. Acta Neuropathol (Berl).

[15] Maynard KR, Collado-Torres L, Weber LM, Uytingco C, Barry BK, Williams SR (2021). Transcriptome-scale spatial gene expression in the human dorsolateral prefrontal cortex. Nat Neurosci.

[16] Guo G, Papanicolaou M, Demarais NJ, Wang Z, Schey KL, Timpson P (2021). Automated annotation and visualisation of high-resolution spatial proteomic mass spectrometry imaging data using HIT-MAP. Nat Commun.

[17] McKhann GM, Knopman DS, Chertkow H, Hyman BT, Jack CR, Kawas CH (2011). The diagnosis of dementia due to Alzheimer’s disease: recommendations from the National Institute on Aging-Alzheimer’s Association workgroups on diagnostic guidelines for Alzheimer’s disease. Alzheimers Dement J Alzheimers Assoc.

[18] Hyman BT, Phelps CH, Beach TG, Bigio EH, Cairns NJ, Carrillo MC (2012). National Institute on Aging-Alzheimer’s Association guidelines for the neuropathologic assessment of Alzheimer’s disease. Alzheimers Dement J Alzheimers Assoc.

[19] Montine TJ, Phelps CH, Beach TG, Bigio EH, Cairns NJ, Dickson DW (2012). National Institute on Aging-Alzheimer’s Association guidelines for the neuropathologic assessment of Alzheimer’s disease: a practical approach. Acta Neuropathol (Berl).

[20] Stack EC, Wang C, Roman KA, Hoyt CC (2014). Multiplexed immunohistochemistry, imaging, and quantitation: a review, with an assessment of Tyramide signal amplification, multispectral imaging and multiplex analysis. Methods San Diego Calif.

[21] Lin J-R, Fallahi-Sichani M, Sorger PK (2015). Highly multiplexed imaging of single cells using a high-throughput cyclic immunofluorescence method. Nat Commun.

[22] Lin J-R, Fallahi-Sichani M, Chen J-Y, Sorger PK (2016). Cyclic immunofluorescence (CycIF), a highly multiplexed method for single-cell imaging. Curr Protoc Chem Biol.

[23] Lin J-R, Izar B, Wang S, Yapp C, Mei S, Shah PM (2018). Highly multiplexed immunofluorescence imaging of human tissues and tumors using t-CyCIF and conventional optical microscopes. Elife.

[24] Schindelin J, Arganda-Carreras I, Frise E, Kaynig V, Longair M, Pietzsch T (2012). Fiji: an open-source platform for biological-image analysis. Nat Methods.

[25] Rueden CT, Schindelin J, Hiner MC, DeZonia BE, Walter AE, Arena ET (2017). Image J2: ImageJ for the next generation of scientific image data. BMC Bioinform.

[26] Du Z, Lin J-R, Rashid R, Maliga Z, Wang S, Aster JC (2019). Qualifying antibodies for image-based immune profiling and multiplexed tissue imaging. Nat Protoc.

[27] Sternberg (1983). Biomedical image processing. Computer.

[28] Dutta A, Zisserman A. The VIA Annotation Software for Images, Audio and Video. Proc 27th ACM Int Conf Multimed. Nice France: ACM; 2019. p. 2276–9. 10.1145/3343031.3350535

[29] Bagwell CB (2005). Hyperlog? A flexible log-like transform for negative, zero, and positive valued data. Cytometry A.

[30] Wang B, Mezlini AM, Demir F, Fiume M, Tu Z, Brudno M (2014). Similarity network fusion for aggregating data types on a genomic scale. Nat Methods.

[31] von Luxburg U (2007). A tutorial on spectral clustering. Stat Comput.

[32] Kuhn M (2008). Building predictive models in *R* using the **caret** package. J Stat Softw.

[33] Greenwell, B, Boehmke, B, Cunningham, J, GBM Developers. Generalized Boosted Regression Models. (2020). https://github.com/gbm-developers/gbm

[34] Friedman JH (2001). Greedy function approximation: a gradient boosting machine. Ann Stat.

[35] Friedman JH (2002). Stochastic gradient boosting. Comput Stat Data Anal.

[36] Paszke A, Gross S, Massa F, Lerer A, Bradbury J, Chanan G, et al. PyTorch: An Imperative Style, High-Performance Deep Learning Library. Wallach H, Larochelle H, Beygelzimer A, Alché-Buc F d\textquotesingle, Fox E, Garnett R, editors. Adv Neural Inf Process Syst [Internet]. (2019);32. https://proceedings.neurips.cc/paper/2019/file/bdbca288fee7f92f2bfa9f7012727740-Paper.pdf

[37] Kingma DP, Ba J. Adam: A Method for Stochastic Optimization. In: Bengio Y, LeCun Y, editors. 3rd Int Conf Learn Represent ICLR 2015 San Diego CA USA May 7–9 2015 Conf Track Proc. 2015. http://arxiv.org/abs/1412.6980

[38] Akiba T, Sano S, Yanase T, Ohta T, Koyama M. Optuna: A Next-generation Hyperparameter Optimization Framework. CoRR. 2019;abs/1907.10902. http://arxiv.org/abs/1907.10902

[39] Bergstra J, Bardenet R, Bengio Y, Kégl B. Algorithms for Hyper-Parameter Optimization. Proc 24th Int Conf Neural Inf Process Syst. Red Hook, NY, USA: Curran Associates Inc.; 2011. pp. 2546–54.

[40] Pedregosa F, Varoquaux G, Gramfort A, Michel V, Thirion B, Grisel O, et al. Scikit-learn: Machine Learning in Python. CoRR. 2012;abs/1201.0490. http://arxiv.org/abs/1201.0490

[41] Fomin V, Anmol J, Desroziers S, Kriss J, Tejani A. High-level library to help with training neural networks in PyTorch. GitHub; 2020. https://github.com/pytorch/ignite

[42] Kokhlikyan N, Miglani V, Martin M, Wang E, Alsallakh B, Reynolds J, et al. Captum: A unified and generic model interpretability library for PyTorch. CoRR. 2020; abs/2009.07896. https://arxiv.org/abs/2009.07896

[43] Simonyan K, Vedaldi A, Zisserman A. Deep Inside Convolutional Networks: Visualising Image Classification Models and Saliency Maps. In: Bengio Y, LeCun Y, editors. 2nd Int Conf Learn Represent ICLR 2014 Banff AB Can April 14–16 2014 Workshop Track Proc. 2014. http://arxiv.org/abs/1312.6034

[44] Sundararajan M, Taly A, Yan Q. Axiomatic attribution for deep networks. Proc 34th Int Conf Mach Learn - Vol 70. JMLR.org; 2017. pp. 3319–28.

[45] Selvaraju RR, Cogswell M, Das A, Vedantam R, Parikh D, Batra D. Grad-CAM: Visual Explanations From Deep Networks via Gradient-Based Localization. Proc IEEE Int Conf Comput Vis ICCV. 2017.

[46] Luke SG (2017). Evaluating significance in linear mixed-effects models in R. Behav Res Methods.

[47] Hopperton KE, Mohammad D, Trépanier MO, Giuliano V, Bazinet RP (2018). Markers of microglia in post-mortem brain samples from patients with Alzheimer’s disease: a systematic review. Mol Psychiatry.

[48] McQuaid S, McConnell R, McMahon J, Herron B (1995). Microwave antigen retrieval for immunocytochemistry on formalin-fixed, paraffin-embedded post-mortem CNS tissue. J Pathol.

[49] Gui Y, Marks JD, Das S, Hyman BT, Serrano-Pozo A (2019). Characterization of the 18 kDa translocator protein (TSPO) expression in post-mortem normal and Alzheimer’s disease brains. Brain Pathol Zurich Switz..

[50] Escartin C, Galea E, Lakatos A, O’Callaghan JP, Petzold GC, Serrano-Pozo A (2021). Reactive astrocyte nomenclature, definitions, and future directions. Nat Neurosci.

[51] Bennett ML, Bennett FC, Liddelow SA, Ajami B, Zamanian JL, Fernhoff NB (2016). New tools for studying microglia in the mouse and human CNS. Proc Natl Acad Sci USA.

[52] Keren-Shaul H, Spinrad A, Weiner A, Matcovitch-Natan O, Dvir-Szternfeld R, Ulland TK (2017). A unique microglia type associated with restricting development of Alzheimer’s disease. Cell.

[53] Lopes KO, Sparks DL, Streit WJ (2008). Microglial dystrophy in the aged and Alzheimer’s disease brain is associated with ferritin immunoreactivity. Glia.

[54] Simpson JE, Ince PG, Lace G, Forster G, Shaw PJ, Matthews F (2010). Astrocyte phenotype in relation to Alzheimer-type pathology in the ageing brain. Neurobiol Aging.

[55] Garcia-Esparcia P, Diaz-Lucena D, Ainciburu M, Torrejón-Escribano B, Carmona M, Llorens F (2018). Glutamate transporter GLT1 expression in Alzheimer disease and dementia with Lewy bodies. Front Aging Neurosci.

[56] Kobayashi E, Nakano M, Kubota K, Himuro N, Mizoguchi S, Chikenji T (2018). Activated forms of astrocytes with higher GLT-1 expression are associated with cognitive normal subjects with Alzheimer pathology in human brain. Sci Rep.

[57] de Rodríguez-Callejas D, Cuervo-Zanatta D, Rosas-Arellano A, Fonta C, Fuchs E, Perez-Cruz C (2019). Loss of ferritin-positive microglia relates to increased iron, RNA oxidation, and dystrophic microglia in the brains of aged male marmosets. Am J Primatol.

[58] Perez-Nievas BG, Stein TD, Tai H-C, Dols-Icardo O, Scotton TC, Barroeta-Espar I (2013). Dissecting phenotypic traits linked to human resilience to Alzheimer’s pathology. Brain J Neurol.

[59] Barroeta-Espar I, Weinstock LD, Perez-Nievas BG, Meltzer AC, Siao TickChong M, Amaral AC (2019). Distinct cytokine profiles in human brains resilient to Alzheimer’s pathology. Neurobiol Dis.

[60] Kyriazis AD, Noroozizadeh S, Refaee A, Choi W, Chu L-T, Bashir A (2019). An end-to-end system for automatic characterization of iba1 immunopositive microglia in whole slide imaging. Neuroinformatics.

[61] Liu M, Ylanko J, Weekman E, Beckett T, Andrews D, McLaurin J (2019). Utilizing supervised machine learning to identify microglia and astrocytes in situ: implications for large-scale image analysis and quantification. J Neurosci Methods.

[62] Morriss NJ, Conley GM, Ospina SM, Meehan Iii WP, Qiu J, Mannix R (2020). Automated quantification of immunohistochemical staining of large animal brain tissue using QuPath software. Neuroscience.

[63] Möhle L, Bascuñana P, Brackhan M, Pahnke J (2021). Development of deep learning models for microglia analyses in brain tissue using DeePathology^TM^ STUDIO. J Neurosci Methods.

[64] Bascuñana P, Brackhan M, Pahnke J (2021). Machine learning-supported analyses improve quantitative histological assessments of amyloid-β deposits and activated microglia. J Alzheimers Dis JAD.

[65] Leyh J, Paeschke S, Mages B, Michalski D, Nowicki M, Bechmann I (2021). Classification of microglial morphological phenotypes using machine learning. Front Cell Neurosci.

[66] Kulkarni PM, Barton E, Savelonas M, Padmanabhan R, Lu Y, Trett K (2015). Quantitative 3-D analysis of GFAP labeled astrocytes from fluorescence confocal images. J Neurosci Methods.

[67] Kayasandik CB, Ru W, Labate D (2020). A multistep deep learning framework for the automated detection and segmentation of astrocytes in fluorescent images of brain tissue. Sci Rep.

