# Supplementary material for: Cyclic multiplex fluorescent immunohistochemistry and machine learning reveal distinct states of astrocytes and microglia in normal aging and Alzheimer’s disease
Source: J Neuroinflammation. 2022 Feb 2;19:30. doi: 10.1186/s12974-022-02383-4 (PMC8808995; doi:10.1186/s12974-022-02383-4)

Supplementary Figure 1. Aβ pathology in the temporal pole cortex.

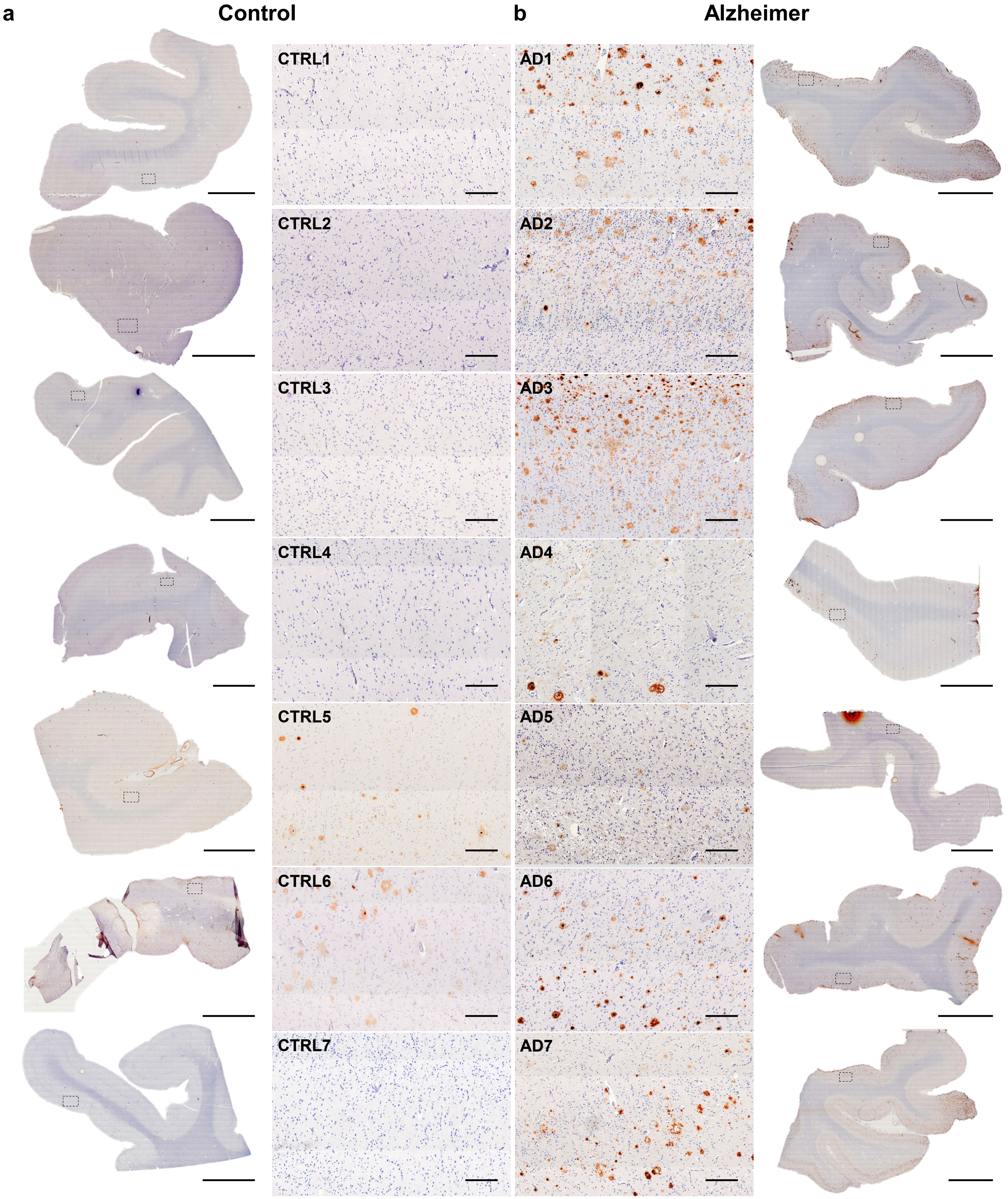

Supplementary Figure 2. Phospho-tau pathology in the temporal pole cortex.

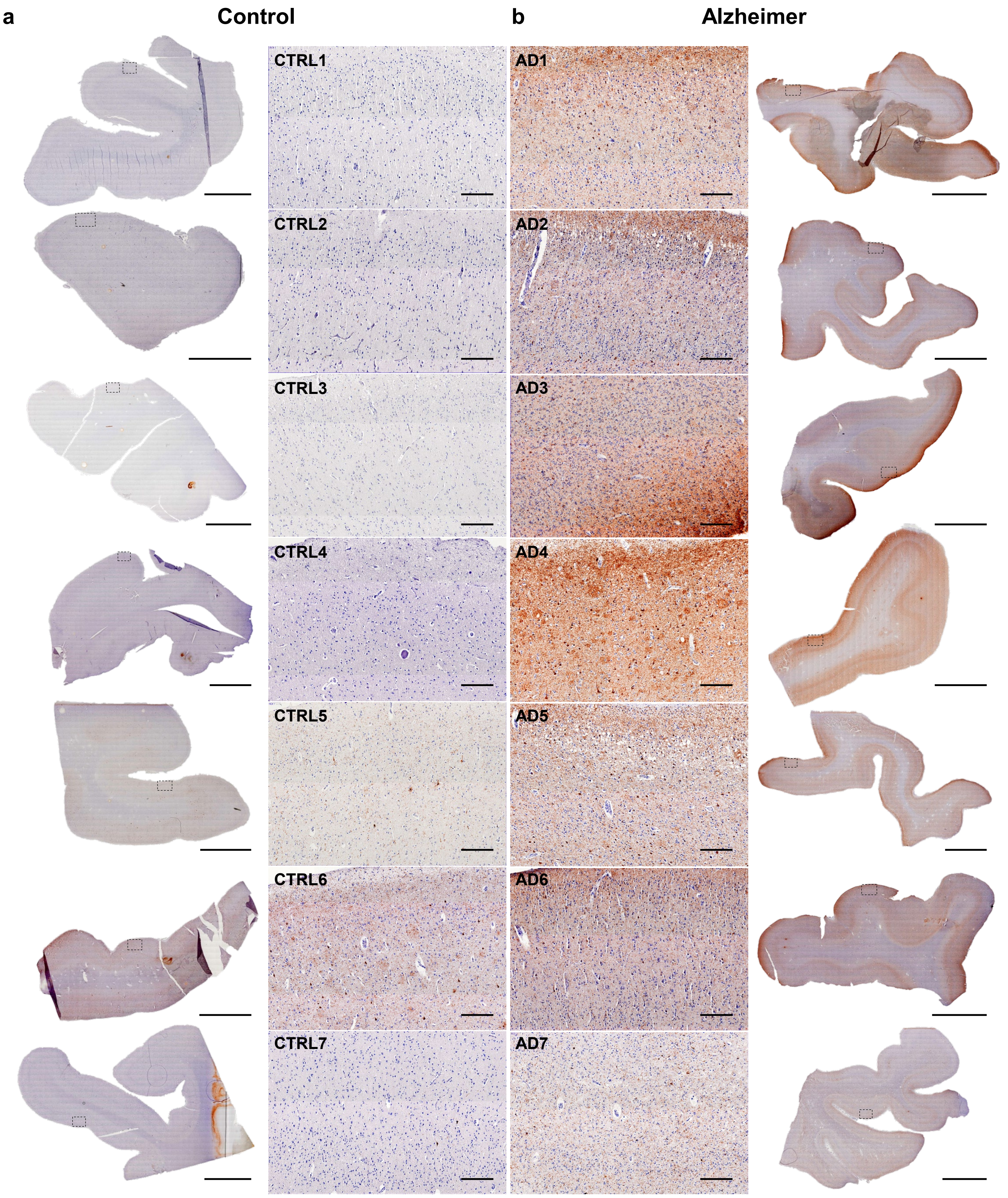

Supplementary Figure 3. Expression levels of selected markers across astrocytic and microglial subclusters from public single-nuclei RNA-seq studies.

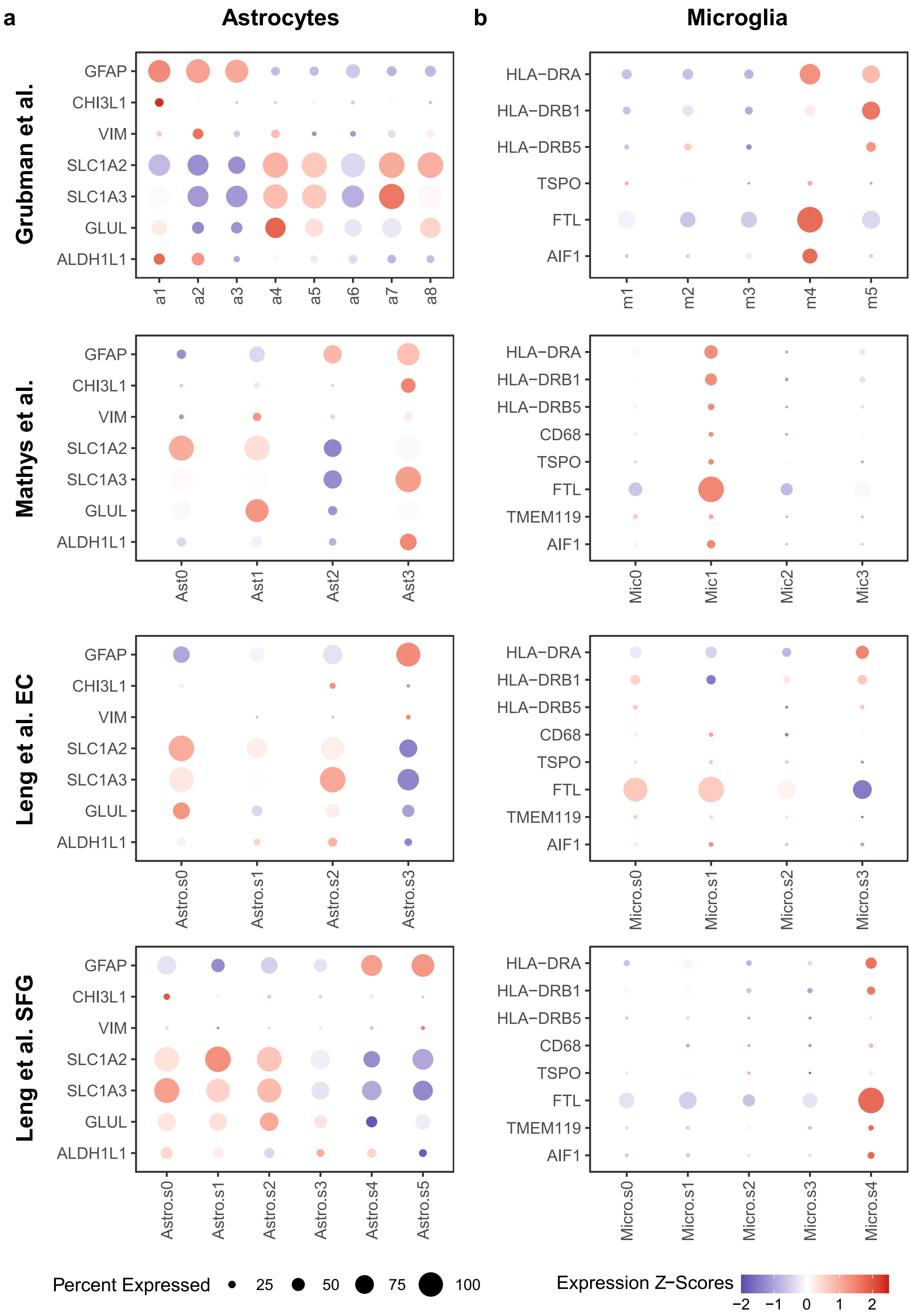

Supplementary Figure 4. Characterization of astrocytes and microglia in AD vs. CTRL by cortical layer.

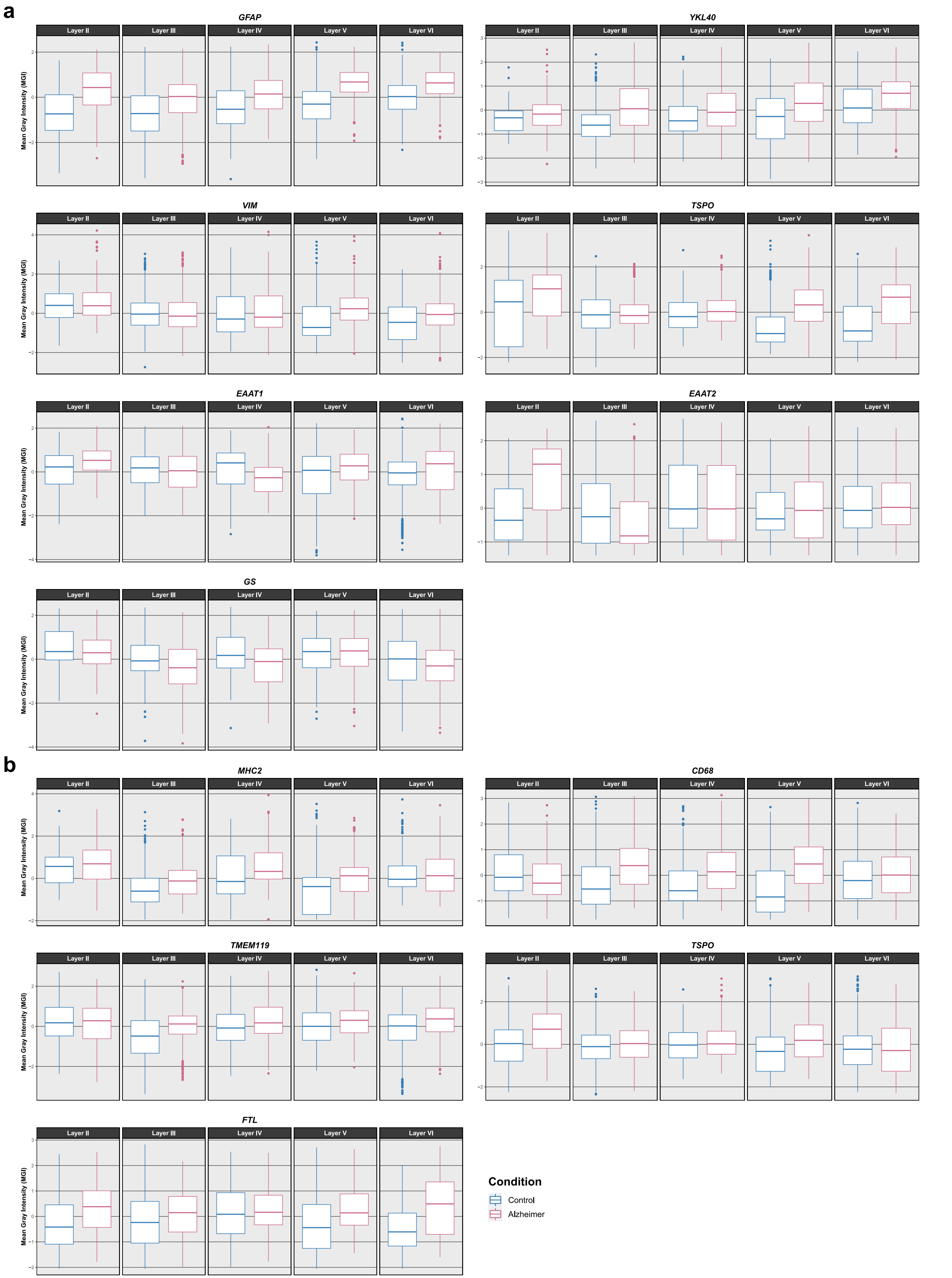

Supplementary Figure 5. Characterization of astrocytic and microglial states by cortical layer.

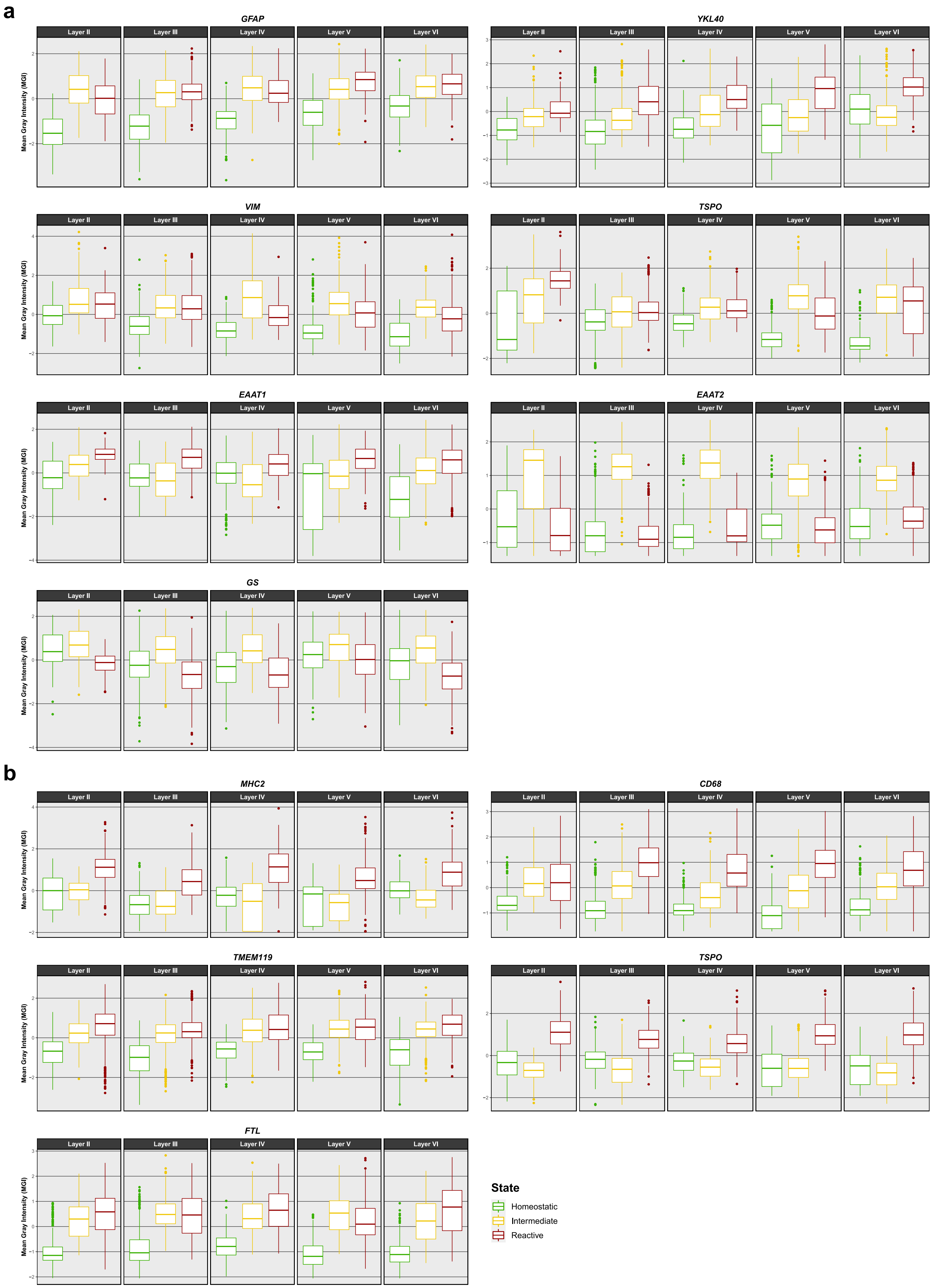

Supplementary Figure 6. Effects of proximity to AD neuropathological changes on astrocyte and microglial phenotypes from two CTRL subjects with abundant Aβ plaques.

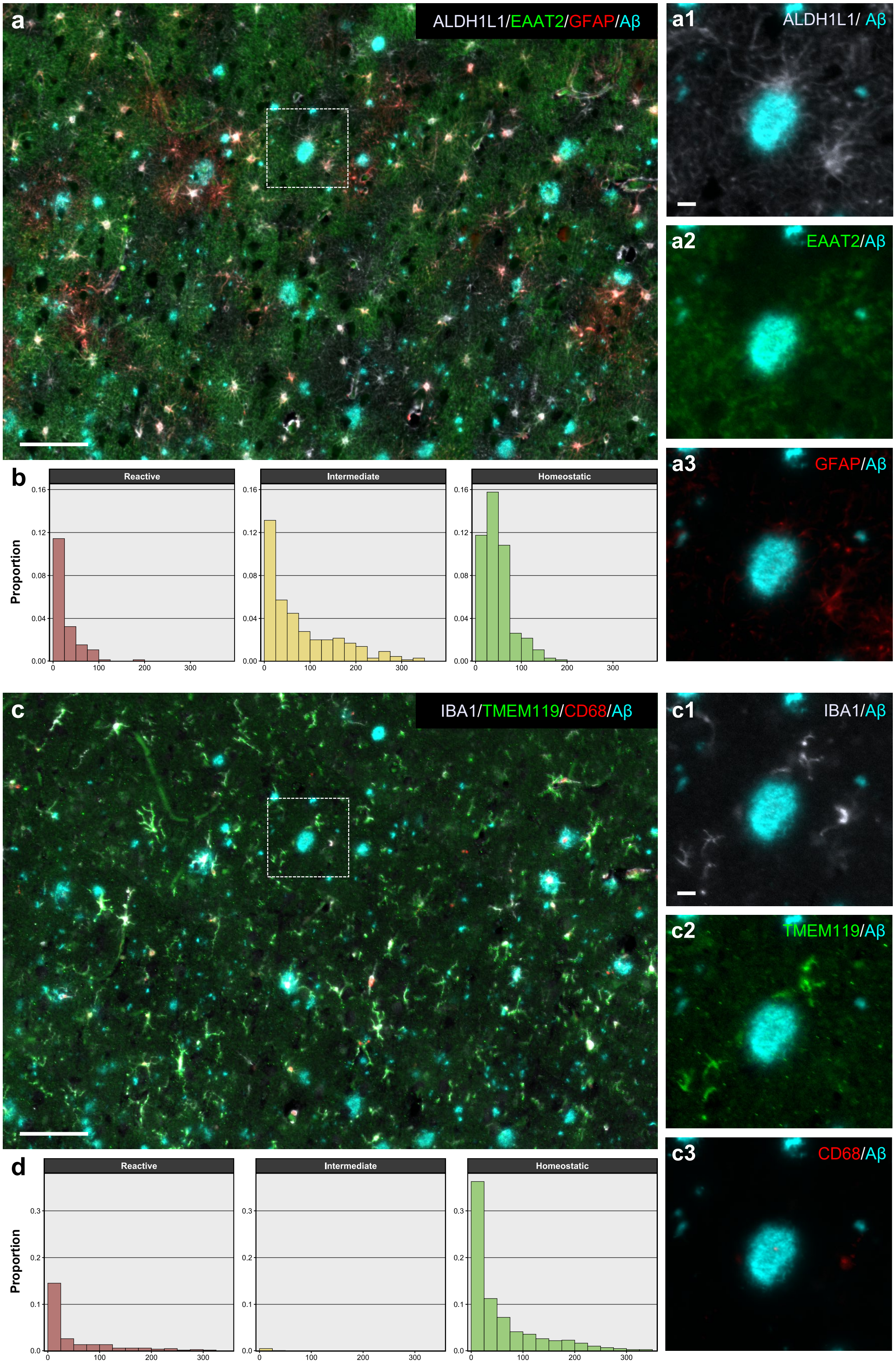

Supplementary Figure 7. Differences in neuritic component of Aβ plaques from CTRL and AD subjects.

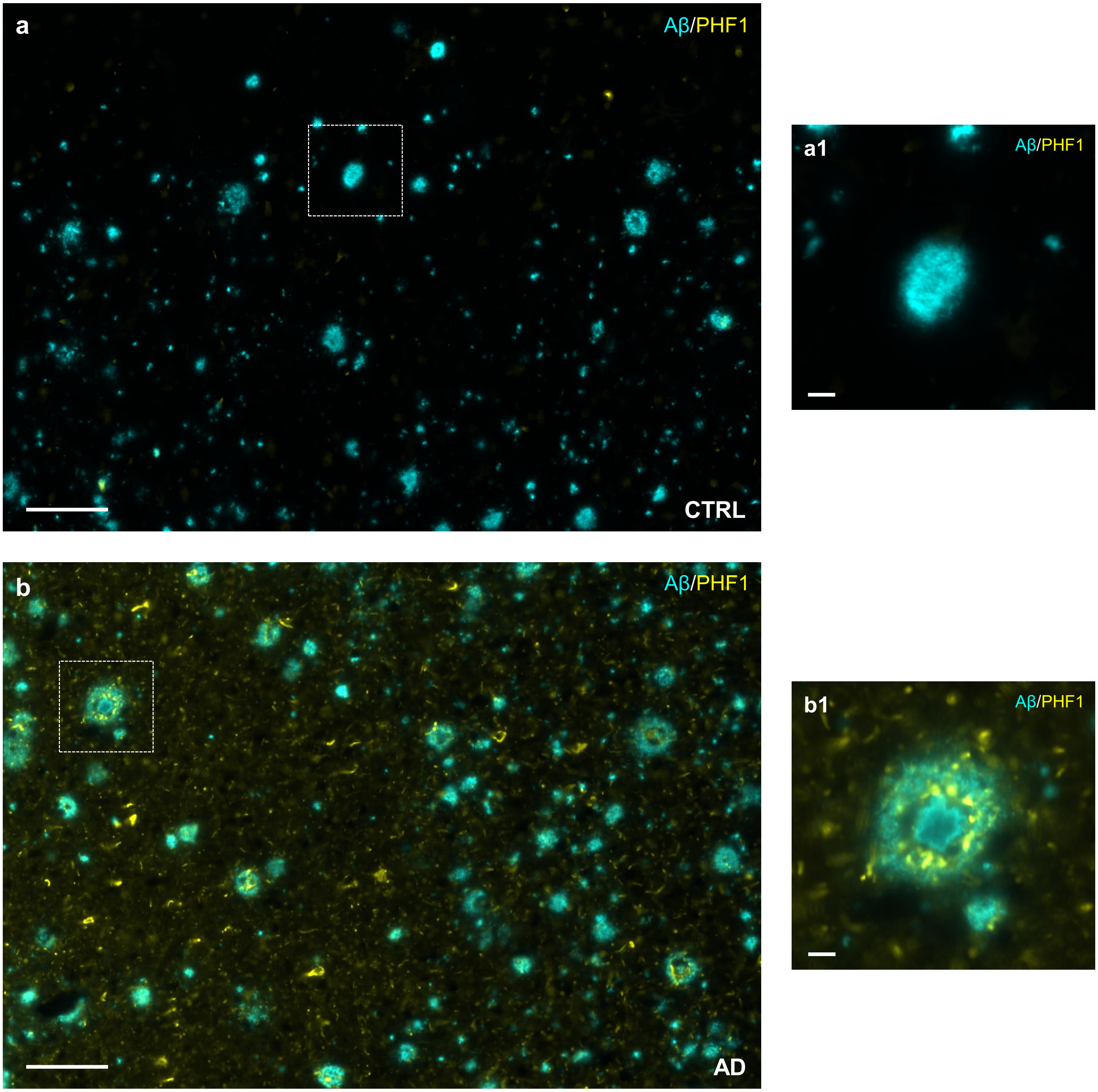

Supplementary Figure 8. Gradient boosting machine models accurately discriminate between glial phenotypes.

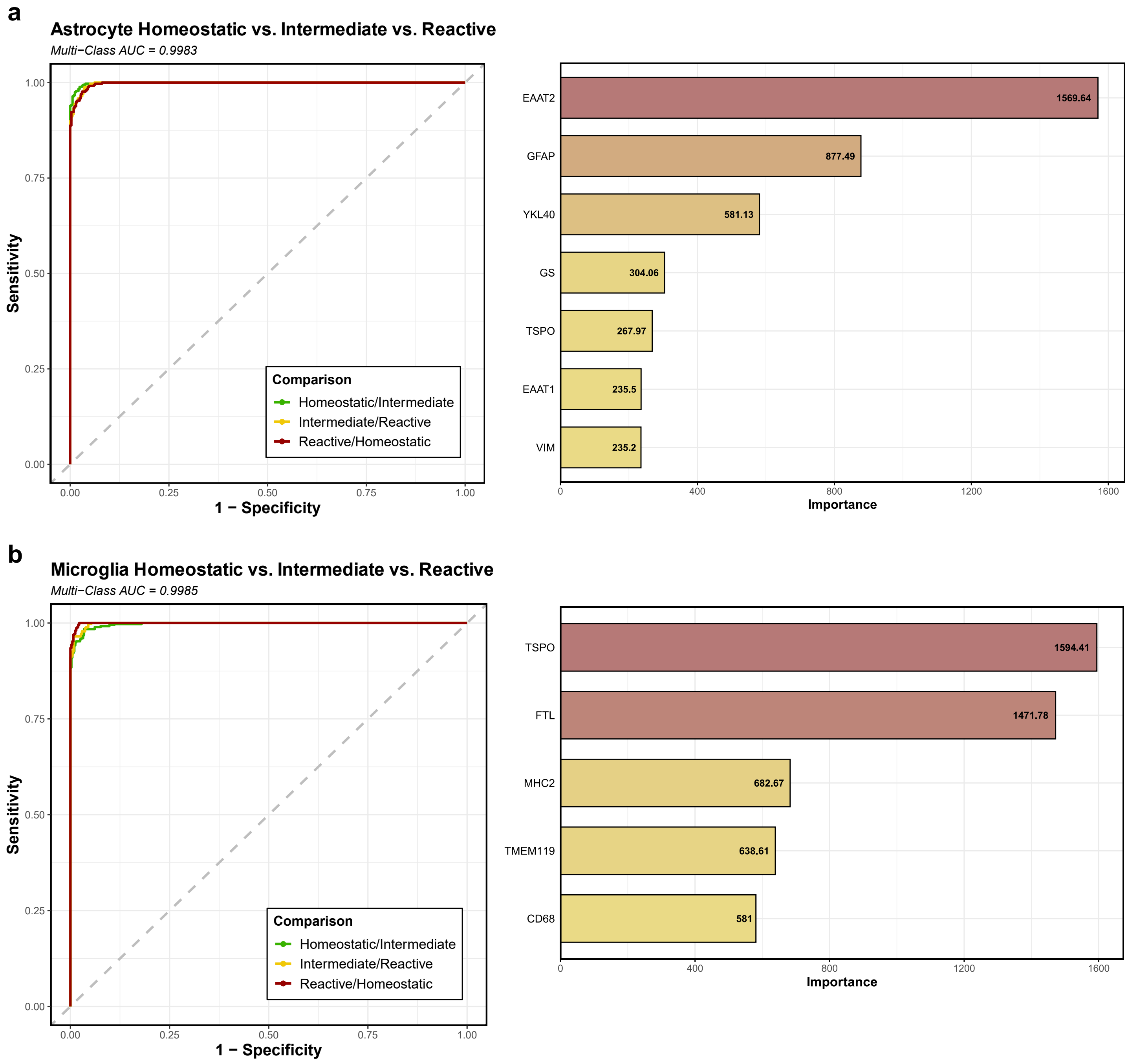

Supplementary Figure 9. Application of deep learning model interpretability functions to astrocytes with extreme classification probabilities.

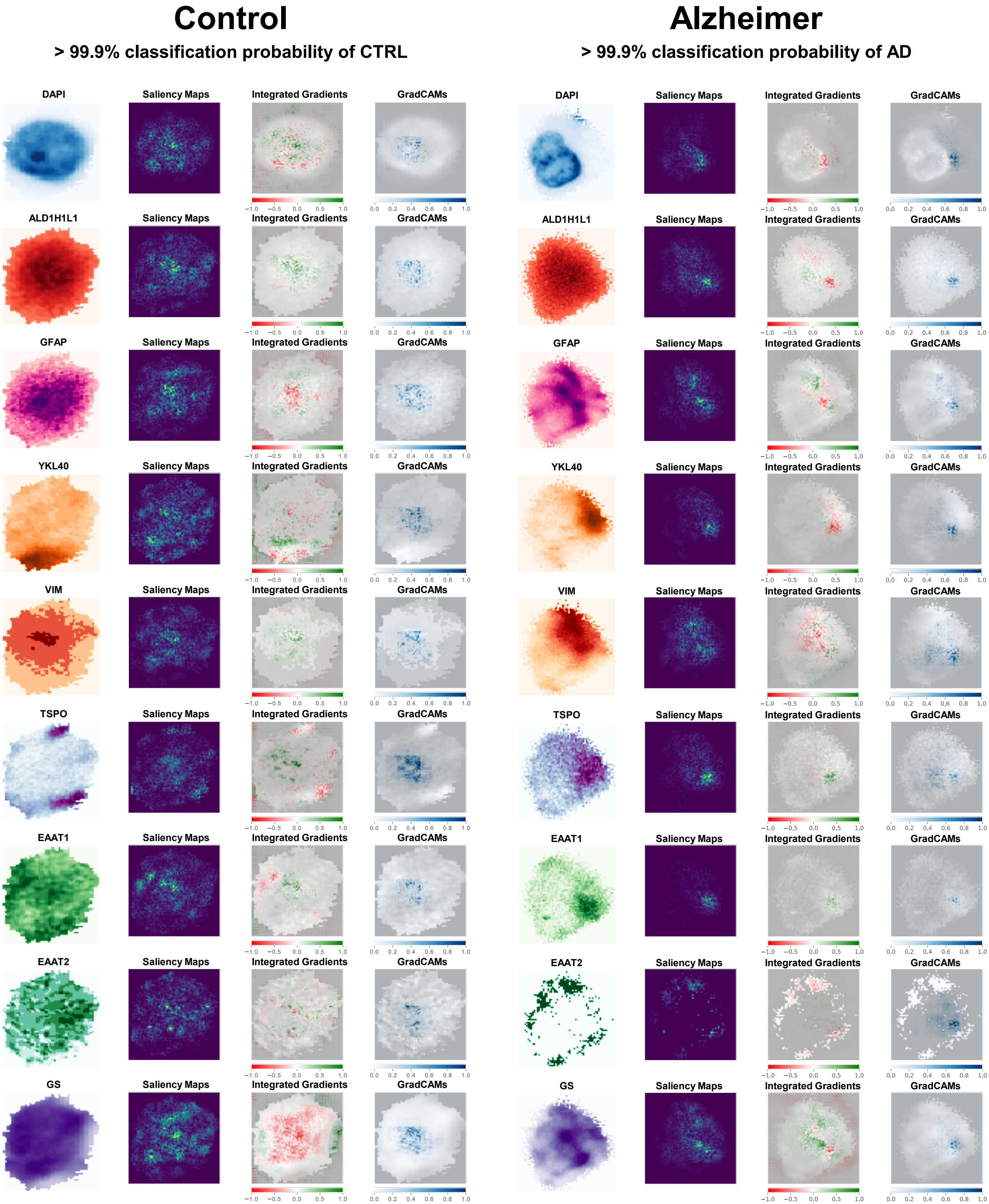

Supplementary Figure 10. Application of deep learning model interpretability functions to microglia with extreme classification probabilities.

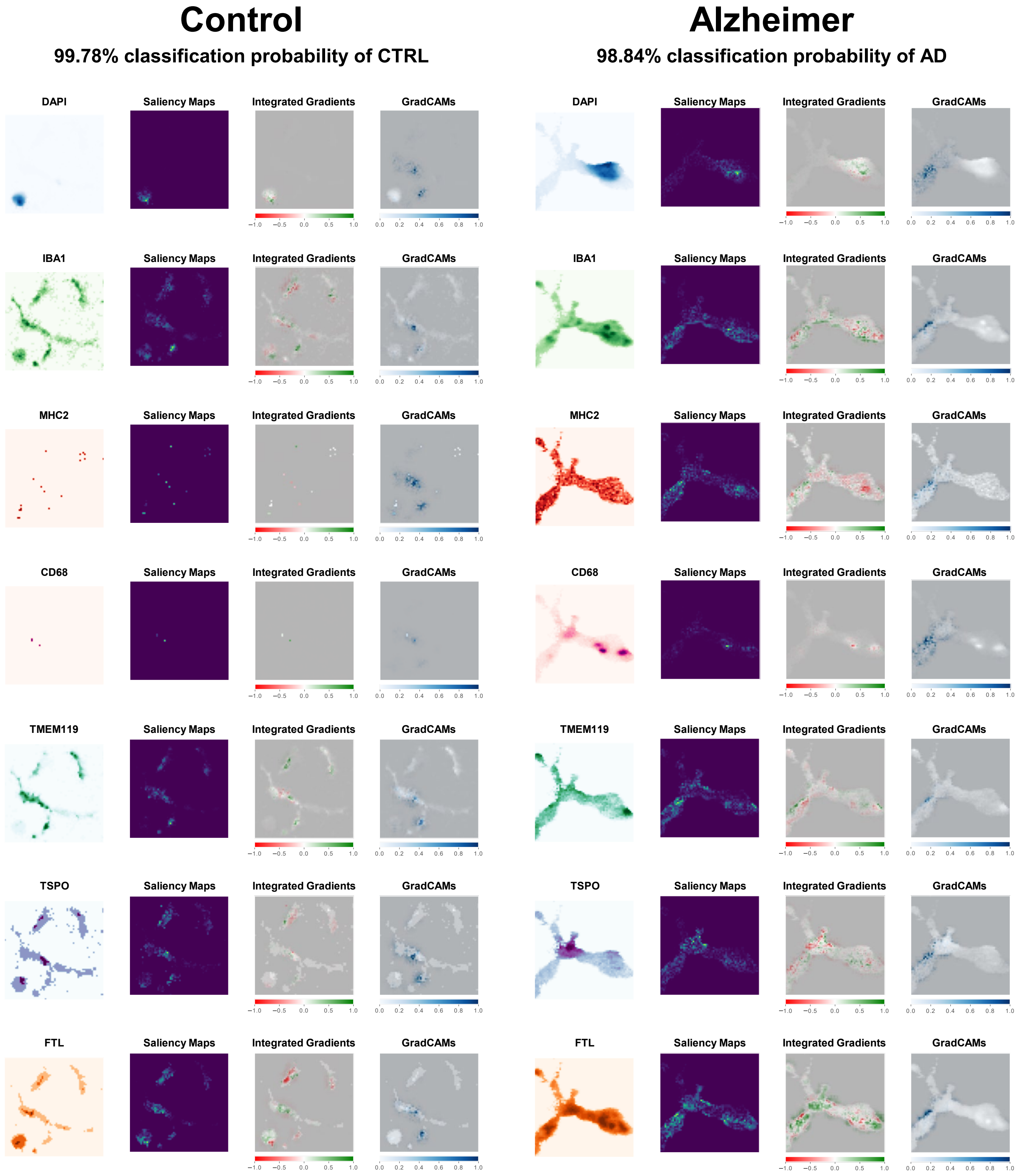

Supplement: Supplementary file 2 — Additional File 2: Figure S1. A β pathology in the temporal pole cortex. Description: Immunohistochemistry for Aβ (mouse monoclonal antibody, clone 6F/3D, Agilent, #M0872, 1:600) with peroxidase/DAB was performed in nearly-adjacent sections to those used for cyclic multiplex fluorescent immunohistochemistry in a Leica BOND-III automated stainer. Sections were counterstained with hematoxylin. Scale bars: 5 mm, insets 200 μm. Figure S2. Phospho-tau pathology in the temporal pole cortex. Description: Immunohistochemsitry for phospho-tauSer202/Thr205(mouse monoclonal antibody, clone AT8, Thermo-Scientific, #MN1020, 1:10,000) with peroxidase/DAB was performed in nearly-adjacent sections to those used for cyclic multiplex fluorescent immunohistochemistry in a Leica BOND-III automated stainer. Sections were counterstained with hematoxylin. Scale bars: 5 mm, insets 200 μm. Figure S3. Expression levels of selected markers across astrocytic and microglial subclusters from public single-nuclei RNA-seq studies. Description: Bubble plots illustrate the percent of nuclei (bubble size) and the gene expression levels (z-scores, color bar) of the astrocytic and microglial markers used in our cyclic multiplex fluorescent immunohistochemistry protocol across the astrocytic and microglial subclusters rendered by several published single-nuclei RNA-seq data sets. Note that our set of markers discriminates some of these transcriptomic subclusters. Figure S4. Characterization of astrocytes and microglia in AD vs. CTRL by cortical layer. Description: Box and whisker plots illustrate the distribution (box: median and interquartile range [IQR]; whiskers: 1.5 × IQR) of mean gray intensity (MGI) z-scores for (a) each astrocytic marker and (b) each microglial marker across the CTRL and AD groups by cortical layer. Only layers II to VI were included in this study. Figure S5. Characterization of astrocytic and microglial states by cortical layer. Description: Box and whisker plots show the distri [file 12974_2022_2383_MOESM2_ESM.pdf]
